# Trends in Oral Anticoagulant Prescribing for Atrial Fibrillation and Active Cancer in UK Primary Care

**DOI:** 10.1016/j.jacadv.2026.103372

**Published:** 2026-10-23

**Authors:** Joseph Junior Damba, Sarah Beradid, Laurent Azoulay, James M. Brophy, Christel Renoux

**Affiliations:** a Centre for Clinical Epidemiology, Lady Davis Institute for Medical Research, Jewish General Hospital, Montréal, Québec, Canada; b Department of Epidemiology, Biostatistics, and Occupational Health, McGill University, Montréal, Québec, Canada; c Gerald Bronfman Department of Oncology, McGill University, Montréal, Québec, Canada; d Division of Cardiology, Department of Medicine, McGill University Health Centre, Montréal, Québec, Canada; e Department of Neurology and Neurosurgery, McGill University, Montréal, Québec, Canada; f Department of Medicine, McGill University, Montréal, Québec, Canada

**Keywords:** apixaban, atrial fibrillation, cancer, edoxaban, oral anticoagulants, prescribing trends

## Abstract

**Background:**

Anticoagulation is essential in patients with atrial fibrillation (AF) and cancer, but recent real-world evidence on oral anticoagulant (OAC) use remains limited.

**Objectives:**

This study aims to describe recent OAC-prescribing trends and identify factors associated with OAC initiation in patients with AF and active cancer in UK primary care.

**Methods:**

Using the Clinical Practice Research Datalink, we identified patients aged ≥50 years with incident AF (cohort entry) between 2010 and 2023 and active cancer diagnosed in the year before cohort entry. We calculated OAC initiation rates in the year following AF diagnosis among eligible patients (CHA_2_DS_2_-VASc ≥2) using Poisson regression, overall and stratified by OAC type, age, sex, and cancer characteristics. Cox proportional hazards regression was used to identify factors associated with OAC initiation.

**Results:**

Among 12,254 eligible patients, 6,940 (56.6%) initiated an OAC. OAC initiation rates rose from 89 per 100 person-years in 2010 to 385 per 100 person-years in 2023 (adjusted rate ratio: 4.32; 95% CI: 3.78-4.93), driven by increased direct oral anticoagulant prescribing. By 2023, apixaban and edoxaban predominated. OAC initiation did not differ by age and sex but was less common in patients with lung cancer (HR: 0.74; 95% CI: 0.67-0.82), metastatic cancer (HR: 0.66; 95% CI: 0.51-0.87), and dementia (HR: 0.75; 95% CI: 0.64-0.88).

**Conclusions:**

In the last decade, OAC initiation has substantially increased among eligible patients with AF and active cancer, driven by direct oral anticoagulant uptake. However, OAC initiation remained lower among patients with lung cancer, metastatic cancer, or specific comorbidities.

Atrial fibrillation (AF) is a common cardiac arrhythmia that is associated with a fivefold increased risk of stroke.^1^ AF is also associated with other age-related comorbidities, including cancer. Cancer is generally considered active when it is recently diagnosed, actively treated, recurrent, locally advanced, or metastatic, whereas a history of cancer refers broadly to any prior cancer diagnosis.^2^, ^3^ Patients with cancer have a 1.5- to 3-fold higher risk of developing AF than the general population, and those with incident AF are also at increased risk of a subsequent cancer diagnosis.^4^, ^5^ This bidirectional association between AF and cancer partly reflects joint pathophysiological mechanisms, such as inflammation and oxidative stress, as well as shared risk factors, including older age, obesity, and smoking.^6^ Patients with AF and cancer have higher risks of stroke and bleeding than those with AF without cancer, complicating anticoagulation decisions.^7^, ^8^

Randomized controlled trials have shown that direct oral anticoagulants (DOACs) are as effective as vitamin K antagonists (VKAs) and are associated with a lower risk of major bleeding in the general population.^9^, ^10^, ^11^, ^12^ In addition, DOACs do not interact with food and require less-intensive monitoring, which makes them more convenient for routine clinical practice. Most AF guidelines now recommend DOACs as the first-line therapy for stroke prevention. However, these guidelines offer no specific anticoagulation recommendations for patients with AF and cancer despite their heightened risks of stroke and bleeding.^13^, ^14^, ^15^

It remains unclear how oral anticoagulants (OACs) are used for stroke prevention in patients with AF and cancer in real-world practice. Prior studies showed that OACs were underutilized in this population, but their short study periods precluded assessment of long-term trends.^16^, ^17^ In addition, DOAC-specific trends in this population have not been described since the latest agent, edoxaban, was approved for stroke prevention.^18^ Monitoring current DOAC-prescribing trends is important, as emerging evidence suggests that individual DOAC agents may differ in their effectiveness and safety profiles.^19^, ^20^, ^21^ Patient characteristics may likewise have evolved since DOACs were first approved, potentially influencing prescribing decisions.^22^

To address these uncertainties, we conducted a population-based cohort study to describe recent prescribing trends of OACs among patients with AF and active cancer in UK primary care between 2010 and 2023. As secondary analyses, we described the clinical characteristics of OAC initiators, identified factors associated with OAC initiation, and assessed persistence with DOACs vs VKAs in this population. We also described prescribing trends in the overall cohort of patients with a history of cancer.

## Methods

### Data source

We used the UK Clinical Practice Research Datalink (CPRD) GOLD and Aurum databases. The CPRD is a large primary care database containing anonymized electronic medical records for more than 60 million patients from over 2,000 practices. The CPRD is broadly representative of the UK population in terms of age, sex, and ethnicity.^23^, ^24^, ^25^ The collected data include patient demographics, lifestyle factors, clinical diagnoses, laboratory test results, prescriptions from general practitioners (GPs), and secondary care referrals. Medical diagnoses and procedures are recorded using the Read Codes and Systematized Nomenclature of Medicine-Clinical Terms (SNOMED-CT) coding systems. Prescriptions written by GPs are automatically captured using a drug dictionary based on the British National Formulary.^23^ Regular quality control procedures are also conducted to verify data accuracy and completeness.^23^, ^26^ The study protocol was approved by the CPRD Research Data Governance (protocol no. 24_004385) and the Research Ethics Board of the Jewish General Hospital, Montréal, Canada.

### Study population

We assembled a cohort of all patients aged ≥50 years with AF diagnosed between January 1, 2010, and December 31, 2023, who also had a diagnosis of cancer (excluding nonmelanoma skin cancer) at or before AF diagnosis. We used relevant cancer codes, which have been validated in CPRD,^27^ and identified the first recorded cancer diagnosis for each patient. Cohort entry was defined as the date of the first AF diagnosis during the study period. We excluded patients with less than 1 year of medical history before cohort entry, those with a prior AF diagnosis to include only incident AF, and those with an OAC prescription in the year before AF diagnosis. Patients with a history of valvular surgery or rheumatic valvular disease at any time before cohort entry were excluded. We further excluded patients with hyperthyroidism or dialysis in the 90 days before cohort entry and those with venous thromboembolism or joint surgery of the hip, femur, or knee in the 30 days before cohort entry. Finally, we excluded patients with a CHA_2_DS_2_-VASc score <2 at cohort entry because they did not meet the guideline-based eligibility criterion for OAC.^13^, ^14^, ^15^ Within this cohort, we identified patients with active cancer whose first recorded cancer diagnosis occurred in the year before cohort entry. Follow-up ended at the earliest of the following: 1 year after cohort entry, death, end of registration in CPRD, or end of the study period (December 31, 2023).

### Oral anticoagulants

We identified all available OACs in the United Kingdom during the study period. OACs included VKAs such as warfarin, phenindione, and acenocoumarol and DOACs including dabigatran, rivaroxaban, edoxaban, and apixaban.

### Statistical analysis

We used Poisson regression to calculate annual incidence rates and 95% CIs for OAC initiation in the year following AF diagnosis, overall and separately for VKAs and each DOAC. OAC incidence rates were stratified by age (50-59, 60-69, 70-79, ≥80), sex (female vs male), cancer type (solid vs hematologic), HAS-BLED (uncontrolled hypertension, abnormal renal or liver function, previous stroke, bleeding history, age >65 years, use of NSAIDs or antiplatelets or alcohol-related disorders) score (<3 vs ≥3), bleeding history (yes vs no), and UK nations (England, Northern Ireland, Scotland, and Wales). We also calculated age- and sex-adjusted rate ratios (aRR) and rate differences comparing each annual OAC incidence rate with that of 2010 (reference year) and the preceding year.

To describe the clinical profile of patients with AF and active cancer who were prescribed OACs in recent years, we restricted the study period to 2015 to 2023, when all DOACs became available in the United Kingdom.^22^, ^28^ We then assessed the following patient characteristics at the time of OAC initiation (detailed description in Supplemental Table 1): patient demographics (age, sex); lifestyle risk factors (body mass index and smoking status within 5 years before OAC initiation); and cancer-related variables (cancer site, presence of metastases, and cancer-related treatments such as radiotherapy, chemotherapy, and surgery). We considered comorbidities measured at any time before OAC initiation, including cardiovascular diseases, prior bleeding, thrombocytopenia, anemia, neurological and psychiatric disorders, history of falls, chronic obstructive pulmonary disease, acute and chronic kidney diseases, liver disease, and diabetes mellitus. We also considered medications used in the year before OAC initiation, including antidiabetics, antihypertensives, antiarrhythmics, digoxin, antiplatelet agents, lipid-lowering drugs, nonsteroidal anti-inflammatory drugs, opioids, systemic corticosteroids, antidepressants, antipsychotics, benzodiazepines and hypnotics, antiepileptics, proton pump inhibitors, H2 blockers, and hormone replacement therapy. We also described OAC dose at initiation (low vs standard doses) separately for each DOAC. Finally, we calculated the CHA_2_DS_2_-VASc (congestive heart failure, hypertension, age ≥75 years, diabetes mellitus, prior stroke/transient ischemic attack, vascular disease, age 65-74 years, and female sex) and modified HAS-BLED scores as measures of stroke and bleeding risks, respectively.^29^, ^30^ The labile international normalized ratio was omitted from the modified HAS-BLED score because of its unavailability. Missing data for body mass index, smoking status, and DOAC dose were coded as an unknown category.

We used a multivariable Cox proportional hazards model conditioning on calendar year to identify factors associated with OAC initiation during the same period. Variables included age, sex, prior stroke/transient ischemic attack, bleeding history, comorbidities (anemia, thrombocytopenia, hypertension, congestive heart failure, diabetes, coronary artery disease, dementia, chronic kidney disease, and liver disease), cancer site (breast, prostate, lung, colorectal, and hematologic cancers), metastatic cancer (yes vs no), and receipt of chemotherapy or radiotherapy. These variables were selected a priori based on prior literature and clinical knowledge.^16^, ^17^, ^31^

For the persistence analysis, patients who initiated OAC within 1 year after AF diagnosis were followed for up to 5 years from their first OAC prescription. Persistence was defined as the time from the first OAC prescription to discontinuation or switch to another OAC class. Patients were considered continuously exposed to DOACs or VKAs if the duration of one prescription overlapped with the date of the subsequent prescription. In the event of nonoverlap, we allowed for a 30-day grace period between 2 successive prescriptions to account for refill delays. We used Kaplan-Meier curves to display persistence over time for DOACs vs VKAs. Finally, we repeated all analyses in the overall cohort of patients with a history of cancer, including a stratified analysis by cancer status (active vs remote, with remote cancer defined as a cancer diagnosis more than 1 year before AF diagnosis).

We conducted three sensitivity analyses to assess the robustness of our results. First, we estimated OAC incidence rates using an alternative active cancer definition based on CPRD codes for cancer diagnosis, receipt of radiotherapy or chemotherapy, cancer-directed surgery, or metastatic cancer in the 6 months before AF diagnosis.^32^ Second, we varied the grace period to 15, 60, and 90 days for the persistence analysis. Third, we repeated the persistence analysis using the Fine and Gray regression model, which accounts for death as a competing risk. Analyses were conducted using SAS version 9.4 (SAS Institute).

## Results

In a cohort of 982,937 patients newly diagnosed with AF between 2010 and 2023, 112,594 (11.4%) had cancer (Supplemental Figure 1), including 14,100 (12.5%) with active cancer. Among the 12,254 (86.9%) patients with active cancer eligible for OAC, 6,940 (56.6%) were newly prescribed an OAC in the year following AF diagnosis, with 5,148 (74.2%) initiating DOACs and 1,792 (25.8%) VKAs. Of the 5,314 (43.4%) OAC noninitiators, 1,740 (32.7%) were prescribed aspirin, and 320 (6.0%) were prescribed low-molecular-weight heparin during the year following AF diagnosis.

Annual OAC initiation rates rose from 89.1 per 100 person-years (PYs) in 2010 to 385.0 per 100 PYs in 2023 (aRR: 4.32; 95% CI: 3.78-4.93) (Supplemental Table 2). Rate differences also showed the same overall pattern (Supplemental Table 3). The increase in OAC initiation was driven by DOAC prescriptions, which rose from 13.2 per 100 PYs in 2013 to 379.4 per 100 PYs in 2023 (aRR: 28.03; 95% CI: 25.56-31.38). By contrast, VKA prescriptions declined steadily from 89.1 per 100 PYs in 2010 to 5.7 per 100 PYs in 2023 (aRR: 0.06; 95% CI: 0.03-0.11) (Figure 1).

**Figure 1:**
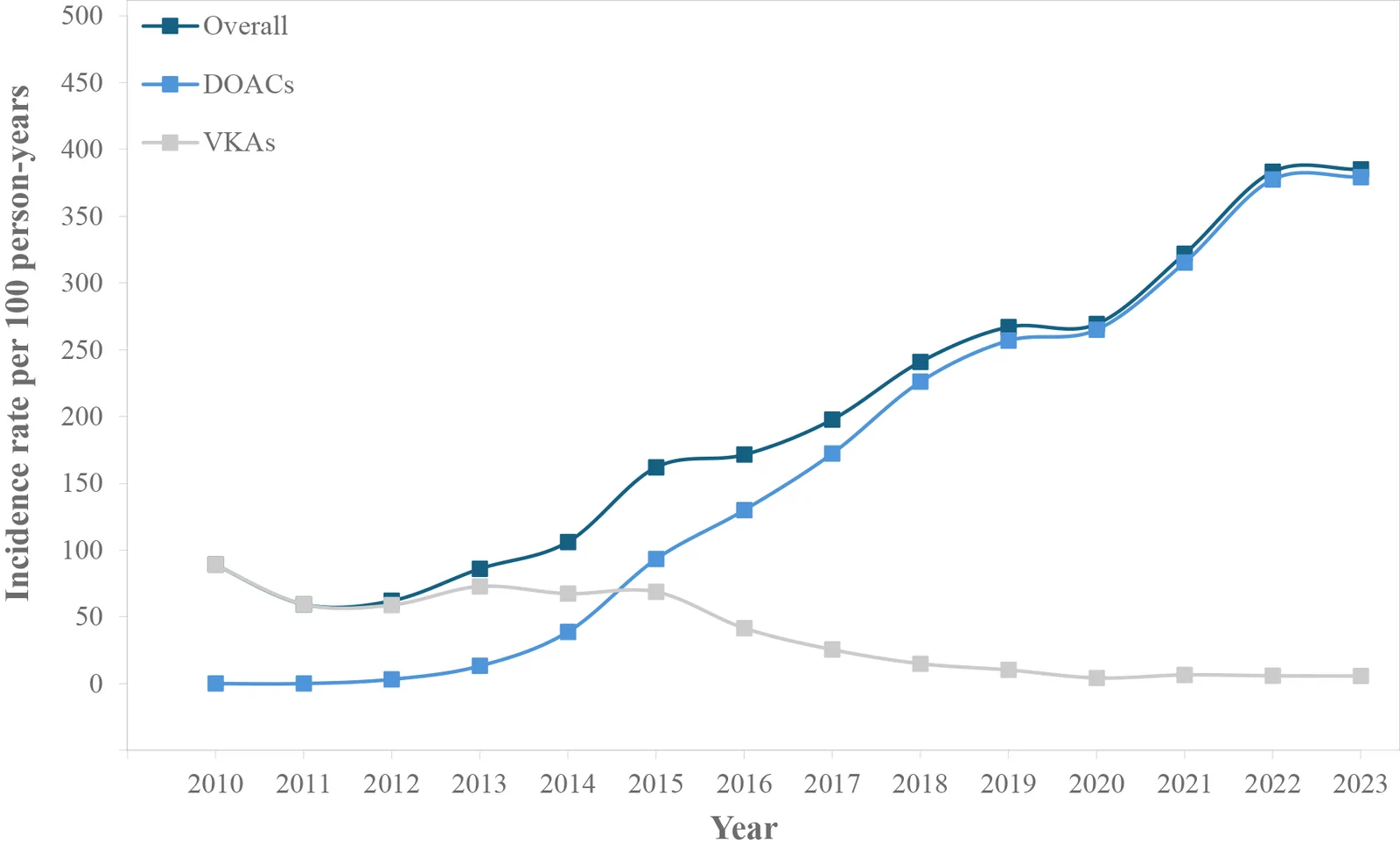
Annual Incidence Rates of Oral Anticoagulant Initiation in Patients With Atrial Fibrillation and Active Cancer in the UK Clinical Practice Research Datalink, 2010 to 2023, Overall and by Oral Anticoagulant Class (DOACs vs VKAs) DOAC = direct oral anticoagulant; VKA = vitamin K antagonist.

Rivaroxaban was the most prescribed DOAC until 2016. Apixaban became the predominant agent from 2017 onwards, with edoxaban ranking second after 2020 (Figure 2). In 2023, apixaban and edoxaban together accounted for 92.8% of OAC prescriptions (51.9% and 40.9%, respectively). The rates of dabigatran initiation were consistently low throughout the study period, peaking at 9.7 per 100 PYs in 2014.

**Figure 2:**
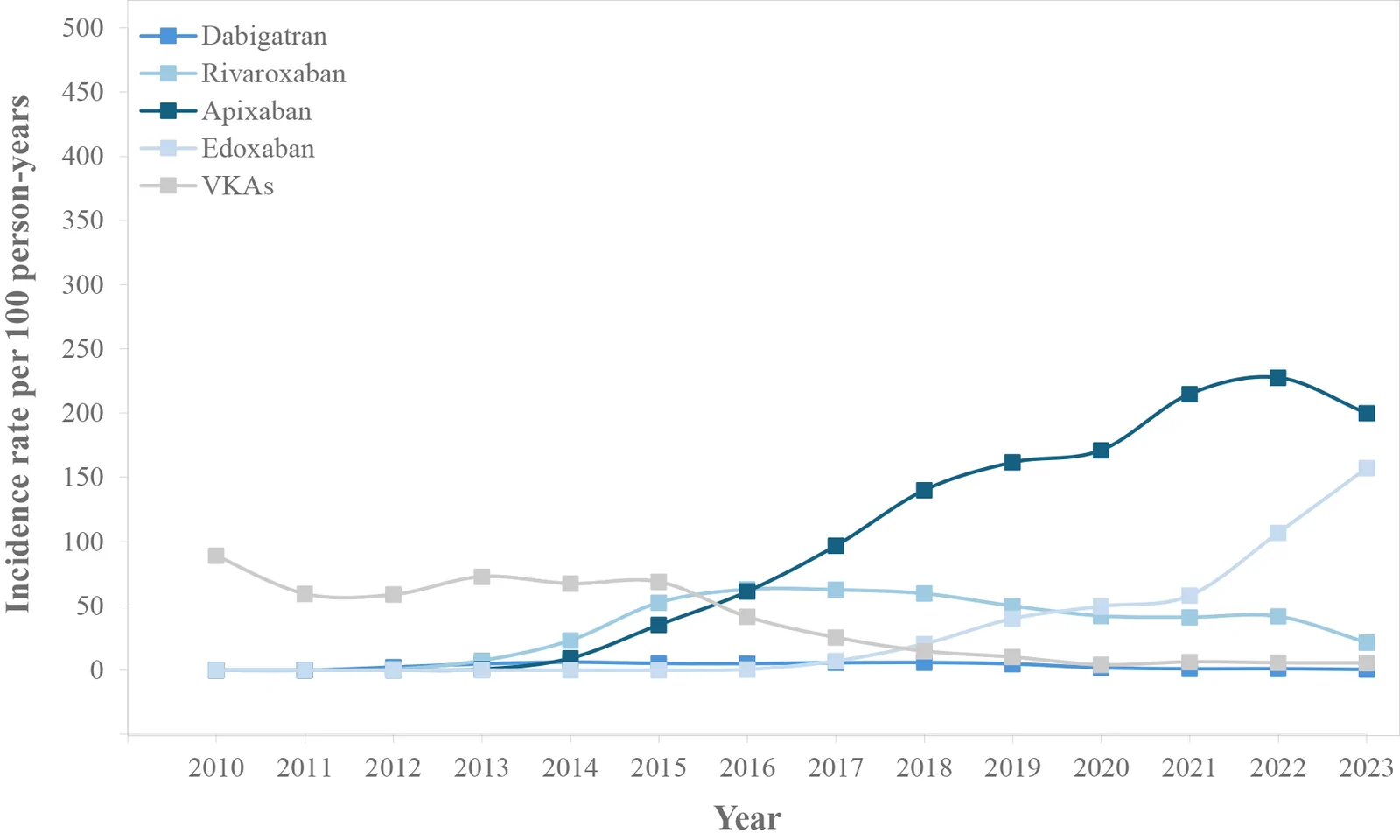
Annual Incidence Rates of Oral Anticoagulant Initiation in Patients With Atrial Fibrillation and Active Cancer in the UK Clinical Practice Research Datalink, 2010 to 2023, by Individual DOACs and VKAs Abbreviations as in Figure 1.

Prescribing trends were consistent across age groups for both DOACs and VKAs, with no clear age-related pattern (Figure 3). Similarly, OAC-prescribing trends were comparable between male and female patients (Supplemental Figure 2). OAC initiation also increased over time across cancer type, HAS-BLED score, and bleeding history strata (Figure 4, Supplemental Figures 3 and 4). Incidence rates were broadly similar between patients with hematologic and solid cancers (Figure 4). However, prescribing patterns varied across UK nations over time. After 2020, apixaban and edoxaban became the most prescribed DOACs in England and Scotland, with relatively similar rates in 2023 (Supplemental Figure 5).

**Figure 3:**
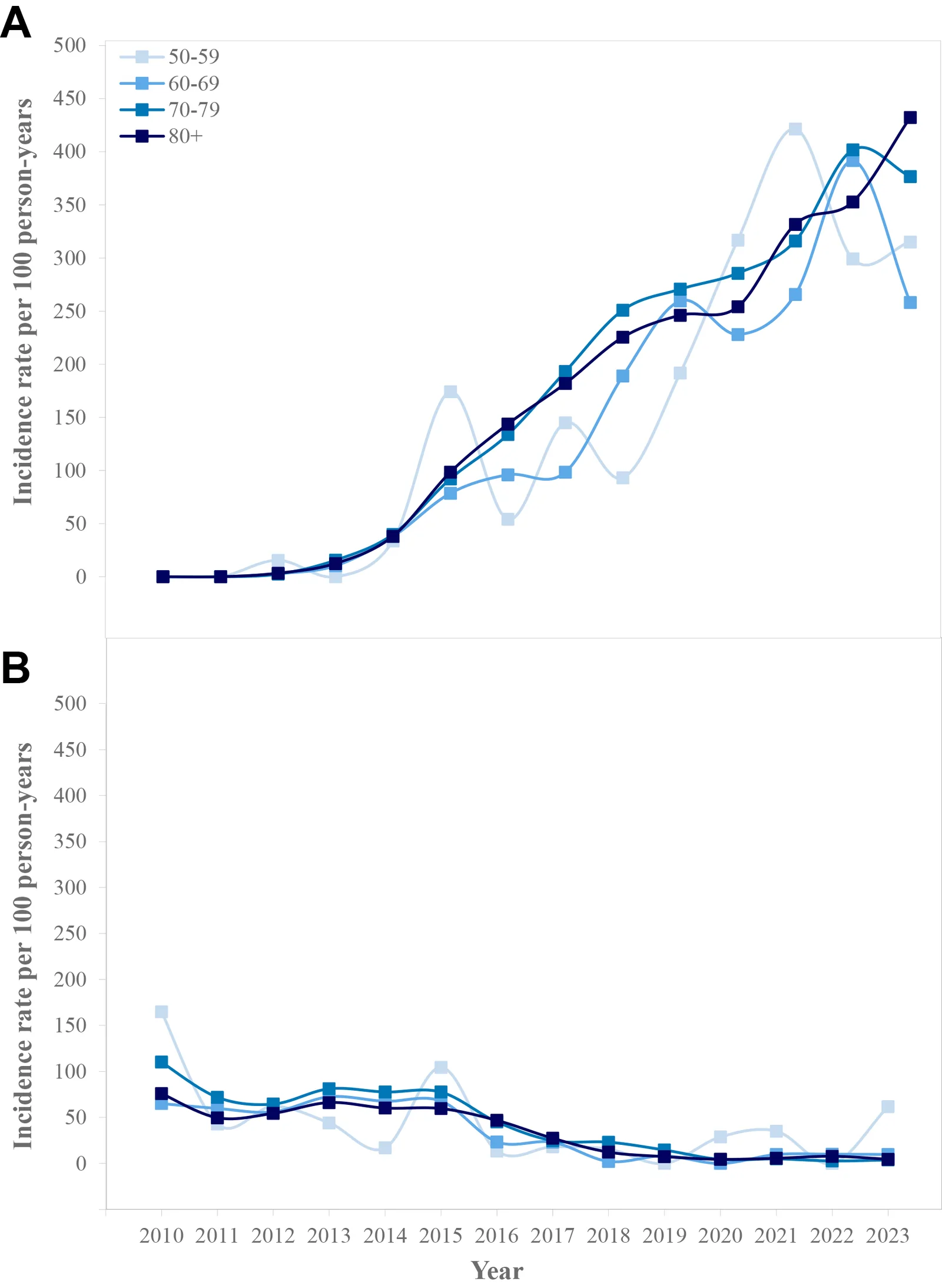
Annual Incidence Rates of Oral Anticoagulant Initiation in Patients With Atrial Fibrillation and Active Cancer in the UK Clinical Practice Research Datalink, 2010 to 2023, Stratified by Age (A) DOACs and (B) VKAs. Abbreviations as in Figure 1.

**Figure 4:**
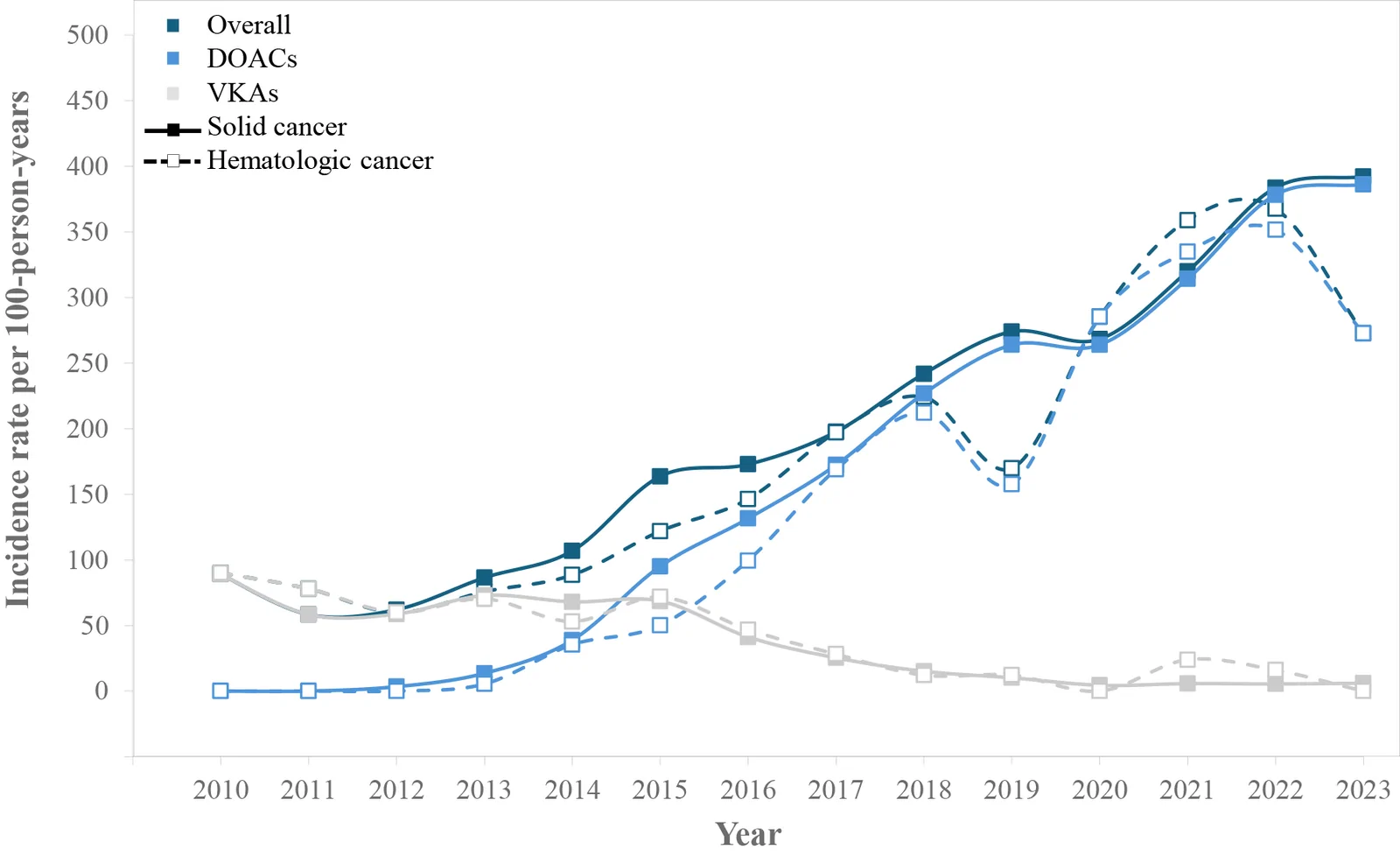
Annual Incidence Rates of Oral Anticoagulant Initiation in Patients With Atrial Fibrillation and Active Cancer in the UK Clinical Practice Research Datalink, 2010 to 2023, Stratified by Cancer Type (Solid vs Hematologic) Abbreviations as in Figure 1.

Table 1 presents the clinical characteristics of patients with active cancer who initiated OACs in the year following AF diagnosis between 2015 and 2023. Overall, patient characteristics were broadly similar across DOACs, although the dabigatran group was small. Rivaroxaban initiators were slightly younger (mean: 77.0 years) than apixaban (78.0) and edoxaban initiators (77.9). Apixaban initiators had slightly more comorbidities than other DOAC initiators, including anemia (20.1%), diabetes (32.9%), and chronic kidney disease (26.0%). VKA initiators had the highest prevalence of chronic kidney disease (31.4%). Across all groups, approximately half of patients had a CHA_2_DS_2_-VASc score of 2 to 3, and more than half had a HAS-BLED score ≥3. Low dose was more common among dabigatran initiators (59.1%), whereas the standard dose was more common among edoxaban (57.8%), apixaban (62.7%), and rivaroxaban (68.3%) initiators.

**Table 1:** Clinical Characteristics of Patients With AF and Active Cancer Who Initiated OACs in UK CPRD, 2015-2023

	Overall^a^ (n = 8,251)	Dabigatran (n = 83)	Rivaroxaban (n = 1,084)	Apixaban (n = 2,879)	Edoxaban (n = 858)	VKAs (n = 490)
Age, y, mean (SD)	78.0 ± 8.1	78.6 ± 7.0	77.0 ± 7.5	78.0 ± 7.8	77.9 ± 8.0	77.6 ± 7.9
Age categories						
50-59 y	124 (1.5)	S^b^	14 (1.3)	42 (1.5)	14 (1.6)	9 (1.8)
60-69 y	1,102 (13.4)	7 (8.4)	168 (15.5)	343 (11.9)	109 (12.7)	64 (13.1)
70-79 y	3,435 (41.6)	34 (41.0)	483 (44.6)	1,241 (43.1)	377 (43.9)	216 (44.1)
≥80 y	3,590 (43.5)	41 (49.4)	419 (38.6)	1,253 (43.5)	358 (41.8)	201 (41.0)
Sex						
Male	4,734 (57.4)	51 (61.4)	607 (56.0)	1,641 (57.0)	501 (58.4)	295 (60.2)
Female	3,517 (42.6)	32 (38.6)	477 (44.0)	1,238 (43.0)	357 (41.6)	195 (39.8)
Smoking status^b^						
Current	1,595 (19.3)	17 (20.5)	174 (16.1)	497 (17.3)	144 (16.8)	96 (19.6)
Former	3,011 (36.5)	27 (32.5)	433 (39.9)	1,036 (36.0)	289 (33.7)	169 (34.5)
Never	2,948 (35.7)	33 (39.8)	410 (37.8)	1,115 (38.7)	323 (37.6)	191 (39.0)
Unknown	697 (8.4)	6 (7.2)	67 (6.2)	231 (8.0)	102 (11.9)	34 (6.9)
BMI						
<18.5 kg/m^2^	153 (1.8)	S^c^	13 (1.2)	52 (1.8)	16 (1.9)	10 (2.0)
18.5-24.9 kg/m^2^	2,014 (24.4)	19 (22.9)	243 (22.4)	665 (23.1)	208 (24.2)	96 (19.6)
25.0-29.9 kg/m^2^	2,536 (30.7)	18 (21.7)	358 (33.0)	926 (32.2)	249 (29.0)	151 (30.8)
≥30.0 kg/m^2^	2,326 (28.2)	27 (32.5)	335 (30.9)	808 (28.1)	274 (31.9)	160 (32.7)
Unknown	1,222 (14.8)	19 (22.9)	135 (12.5)	428 (14.9)	111 (12.9)	73 (14.9)
Comorbidities^d^						
Thrombocytopenia	23 (0.3)	S^b^	S^b^	6 (0.2)	S^b^	S^b^
Anemia	1,701 (20.6)	15 (18.1)	174 (16.1)	579 (20.1)	159 (18.5)	106 (21.6)
Prior bleeding	3,182 (38.6)	37 (44.6)	413 (38.1)	1,107 (38.4)	335 (39.0)	194 (39.6)
Peripheral artery disease	168 (2.0)	S^b^	26 (2.4)	96 (3.3)	18 (2.1)	12 (2.4)
Stroke/TIA	1,189 (14.4)	16 (19.3)	137 (12.6)	486 (16.9)	116 (13.5)	76 (15.5)
Systemic embolism	172 (2.1)	S^b^	51 (4.7)	77 (2.7)	15 (1.7)	16 (3.3)
Coronary artery disease	1,575 (19.1)	15 (18.1)	217 (20.0)	529 (18.4)	151 (17.6)	97 (19.8)
Myocardial infarction	895 (10.8)	6 (7.2)	127 (11.7)	298 (10.4)	81 (9.4)	47 (9.6)
Congestive heart failure	1,017 (12.3)	16 (19.3)	128 (11.8)	435 (15.1)	114 (13.3)	80 (16.3)
Hypertension	5,727 (69.4)	59 (71.1)	747 (68.9)	2,037 (70.8)	612 (71.3)	356 (72.7)
CABG-PCI	683 (8.3)	8 (9.6)	91 (8.4)	252 (8.8)	62 (7.2)	49 (10.0)
Pacemaker	326 (4.0)	S^b^	44 (4.1)	112 (3.9)	54 (6.3)	28 (5.7)
History of falls	1,903 (23.1)	22 (26.5)	218 (20.1)	659 (22.9)	179 (20.9)	101 (20.6)
COPD	2,176 (26.4)	21 (25.3)	284 (26.2)	770 (26.7)	197 (23.0)	117 (23.9)
Acute kidney injury	586 (7.1)	S^b^	49 (4.5)	235 (8.2)	52 (6.1)	32 (6.5)
Chronic kidney disease	2,167 (26.3)	19 (22.9)	248 (22.9)	749 (26.0)	208 (24.2)	154 (31.4)
Depression	1,765 (21.4)	21 (25.3)	232 (21.4)	656 (22.8)	194 (22.6)	102 (20.8)
Dementia or MCI	658 (8.0)	S^b^	52 (4.8)	108 (3.8)	28 (3.3)	14 (2.9)
Parkinson disease	17 (0.2)	S^b^	S^b^	6 (0.2)	S^b^	S^b^
Liver disease	423 (5.1)	S^b^	58 (5.4)	158 (5.5)	50 (5.8)	14 (2.9)
Diabetes mellitus	2,626 (31.8)	22 (26.5)	332 (30.6)	946 (32.9)	262 (30.5)	160 (32.7)
Comedications^e^						
Antidiabetic	1,455 (17.6)	17 (20.5)	179 (16.5)	534 (18.6)	128 (14.9)	92 (18.8)
Antihypertensive drugs	6,188 (75.0)	68 (81.9)	871 (80.4)	2,329 (80.9)	685 (79.8)	413 (84.3)
Lipid-lowering drugs	3,899 (47.2)	45 (54.2)	540 (49.8)	1,391 (48.3)	425 (49.5)	230 (46.9)
Antiarrhythmics	77 (0.9)	S^b^	20 (1.8)	38 (1.3)	11 (1.3)	11 (2.2)
Digoxin	90 (1.1)	S^b^	48 (4.4)	92 (3.2)	24 (2.8)	26 (5.3)
Antiplatelets	3,102 (37.6)	38 (45.8)	412 (38.0)	1,119 (38.9)	285 (33.2)	217 (44.3)
NSAIDs	1,687 (20.4)	17 (20.5)	258 (23.8)	574 (19.9)	161 (18.8)	115 (23.5)
Antidepressants	1,355 (16.4)	15 (18.1)	179 (16.5)	483 (16.8)	136 (15.9)	79 (16.1)
Antipsychotics	512 (6.2)	S^b^	65 (6.0)	163 (5.7)	41 (4.8)	31 (6.3)
Antiepileptics	718 (8.7)	S^b^	81 (7.5)	272 (9.4)	81 (9.4)	42 (8.6)
Benzodiazepines/hypnotics	557 (6.8)	S^b^	61 (5.6)	190 (6.6)	56 (6.5)	26 (5.3)
Systemic corticosteroids	1,369 (16.6)	11 (13.3)	179 (16.5)	447 (15.5)	97 (11.3)	67 (13.7)
Opioids	3,267 (39.6)	34 (41.0)	392 (36.2)	1,155 (40.1)	288 (33.6)	204 (41.6)
H2 blockers	299 (3.6)	S^b^	44 (4.1)	105 (3.6)	20 (2.3)	20 (4.1)
Proton pump inhibitors	4,154 (50.3)	32 (38.6)	546 (50.4)	1,465 (50.9)	427 (49.8)	245 (50.0)
Hormone replacement therapy	116 (1.4)	S^b^	15 (1.4)	42 (1.5)	11 (1.3)	9 (1.8)
Cancer type						
Lung	797 (9.7)	7 (8.4)	89 (8.2)	238 (8.3)	62 (7.2)	25 (5.1)
Prostate	679 (8.2)	7 (8.4)	122 (11.3)	272 (9.4)	104 (12.1)	55 (11.2)
Colorectal	703 (8.5)	9 (10.8)	80 (7.4)	228 (7.9)	85 (9.9)	37 (7.6)
Breast	513 (6.2)	10 (12.0)	99 (9.1)	201 (7.0)	68 (7.9)	32 (6.5)
Hematologic	380 (4.6)	S^b^	45 (4.2)	141 (4.9)	28 (3.3)	27 (5.5)
Other cancers	5,179 (62.8)	50 (60.2)	649 (59.9)	1,799 (62.5)	511 (59.6)	314 (64.1)
Metastatic cancer	109 (1.3)	S^b^	6 (0.6)	38 (1.3)	6 (0.7)	S^b^
Cancer-related treatment						
Radiotherapy	303 (3.7)	S^b^	39 (3.6)	129 (4.5)	41 (4.8)	24 (4.9)
Chemotherapy	447 (5.4)	7 (8.4)	51 (4.7)	159 (5.5)	51 (5.9)	22 (4.5)
Surgery	273 (3.3)	6 (7.2)	38 (3.5)	129 (4.5)	49 (5.7)	13 (2.7)
CHA_2_DS_2_-VASc						
2-3	4,124 (50.0)	34 (41.0)	542 (50.0)	1,345 (46.7)	444 (51.7)	236 (48.2)
4-5	3,300 (40.0)	39 (47.0)	465 (42.9)	1,199 (41.6)	327 (38.1)	194 (39.6)
≥6	827 (10.0)	10 (12.0)	77 (7.1)	335 (11.6)	87 (10.1)	60 (12.2)
Modified HAS-BLED						
<3	2,956 (35.8)	28 (33.7)	469 (43.3)	1,140 (39.6)	363 (42.3)	175 (35.7)
≥3	5,295 (64.2)	55 (66.3)	615 (56.7)	1,739 (60.4)	495 (57.7)	315 (64.3)
Dose						
Low dose	-	49 (59.0)	134 (12.4)	638 (22.2)	219 (25.5)	-
Standard dose	-	25 (30.1)	740 (68.3)	1,805 (62.7)	496 (57.8)	-
Unknown	-	9 (10.8)	210 (19.4)	436 (15.1)	143 (16.7)	-

Values are n (%), unless otherwise indicated.

AF = atrial fibrillation; BMI = body mass index; CABG-PCI = coronary artery bypass surgery or percutaneous coronary intervention; COPD = chronic obstructive pulmonary disease; CPRD = Clinical Practice Research Datalink; MCI = mild cognitive impairment; NSAID = nonsteroidal anti-inflammatory drug; OAC = oral anticoagulant; TIA = transient ischemic attack; UK = United Kingdom; VKA = vitamin K antagonist.

a Clinical characteristics for the overall cohort are measured at AF diagnosis.

b Smoking and BMI were measured in the 5 years prior to OAC initiation.

c Comorbidities were measured at anytime prior to OAC initiation.

d Cell counts <5 are suppressed, in accordance with CPRD confidentiality policy.

e Medications were measured in the year prior to OAC initiation.

Table 2 presents the factors associated with OAC initiation in the year following AF diagnosis between 2015 and 2023. OAC initiation was associated with prior stroke and solid cancers (breast and prostate). By contrast, inverse associations were observed for lung cancer, metastatic cancer, chemotherapy, anemia, and dementia.

**Table 2:** Factors Associated With OAC Initiation in the Year Following AF Diagnosis in Patients With Active Cancer in UK CPRD, 2015-2023^a^

	Initiators (n = 5,394)	Noninitiators (n, = 2,857)	Crude HR (95% CI)	Adjusted^b^ HR (95% CI)
Age, y				
60-69	691 (12.8)	411 (14.4)	0.96 (0.76-1.21)	0.96 (0.74-1.23)
70-79	2,351 (43.6)	1,084 (37.9)	1.11 (0.89-1.39)	1.17 (0.91-1.49)
≥80	2,272 (42.1)	1,318 (46.1)	1.04 (0.83-1.30)	1.08 (0.85-1.39)
Male sex	3,095 (57.4)	1,639 (57.4)	1.00 (0.96-1.06)	0.97 (0.90-1.04)
Prior stroke/TIA	798 (14.8)	391 (13.7)	1.12 (1.04-1.21)	1.19 (1.09-1.29)
Bleeding history	2,062 (38.2)	1,120 (39.2)	0.95 (0.90-1.00)	0.94 (0.88-0.99)
Comorbidities				
Anemia	997 (18.5)	704 (24.6)	0.80 (0.75-0.87)	0.83 (0.77-0.90)
Thrombocytopenia	10 (0.2)	13 (0.5)	0.58 (0.32-1.04)	0.60 (0.33-1.10)
Dementia	191 (3.5)	467 (5.8)	0.74 (0.64-0.86)	0.75 (0.64-0.88)
Chronic kidney disease	1,378 (25.5)	789 (27.6)	0.97 (0.91-1.03)	0.97 (0.90-1.03)
Hypertension	3,796 (70.4)	1,931 (67.6)	1.08 (1.01-1.14)	1.06 (0.99-1.14)
Congestive heart failure	675 (12.5)	342 (12.0)	1.05 (0.96-1.13)	1.06 (0.97-1.16)
Coronary artery disease	995 (18.4)	580 (20.3)	0.98 (0.92-1.05)	0.98 (0.91-1.06)
Diabetes	1,705 (31.6)	921 (32.2)	0.98 (0.93-1.04)	1.00 (0.94-1.07)
Liver disease	281 (5.2)	142 (5.0)	0.98 (0.87-1.11)	1.04 (0.91-1.18)
Cancer site				
Lung	421 (7.8)	376 (13.2)	0.75 (0.68-0.83)	0.74 (0.67-0.82)
Breast	410 (7.6)	103 (3.6)	1.38 (1.24-1.52)	1.31 (1.17-1.46)
Colorectal	439 (8.1)	264 (9.2)	0.83 (0.75-0.91)	0.83 (0.76-0.93)
Prostate	560 (10.4)	119 (4.2)	1.56 (1.43-1.70)	1.45 (1.32-1.60)
Hematologic	244 (4.5)	136 (4.8)	0.87 (0.77-0.98)	0.88 (0.78-1.00)
Metastatic cancer	53 (1.0)	56 (2.0)	0.73 (0.55-0.95)	0.66 (0.51-0.87)
Cancer treatment				
Radiotherapy	199 (3.7)	104 (3.6)	1.01 (0.88-1.16)	1.10 (0.94-1.28)
Chemotherapy	251 (4.7)	196 (6.9)	0.71 (0.62-0.80)	0.76 (0.66-0.87)
CHA_2_DS_2_-VASc^c^				
4-5	2,405 (44.6)	895 (31.3)	1.12 (1.06-1.18)	
≥6	530 (9.8)	297 (10.4)	1.08 (0.98-1.18)	
Modified HAS-BLED^d^ ≥3	3,490 (64.7)	1805 (63.2)	1.08 (1.02-1.15)	

Abbreviations as in Table 1.

a Comorbidities were measured at any time before AF diagnosis. Cancer treatment was measured in the year before AF diagnosis.

b Estimates represent the association between each variable and OAC initiation, adjusted for all other variables listed in the table.

c Estimates for the CHA_2_DS_2_-VASc categories were calculated in a separate model.

d Estimate for the HAS-BLED score was calculated in a separate model.

Persistence was higher with DOACs than with VKAs, with both curves diverging approximately 3 months after treatment initiation (Figure 5). The median treatment persistence was 44.7 (95% CI: 42.5-49.0) months for DOACs and 15.6 (95% CI: 14.1-16.9) months for VKAs.

**Figure 5:**
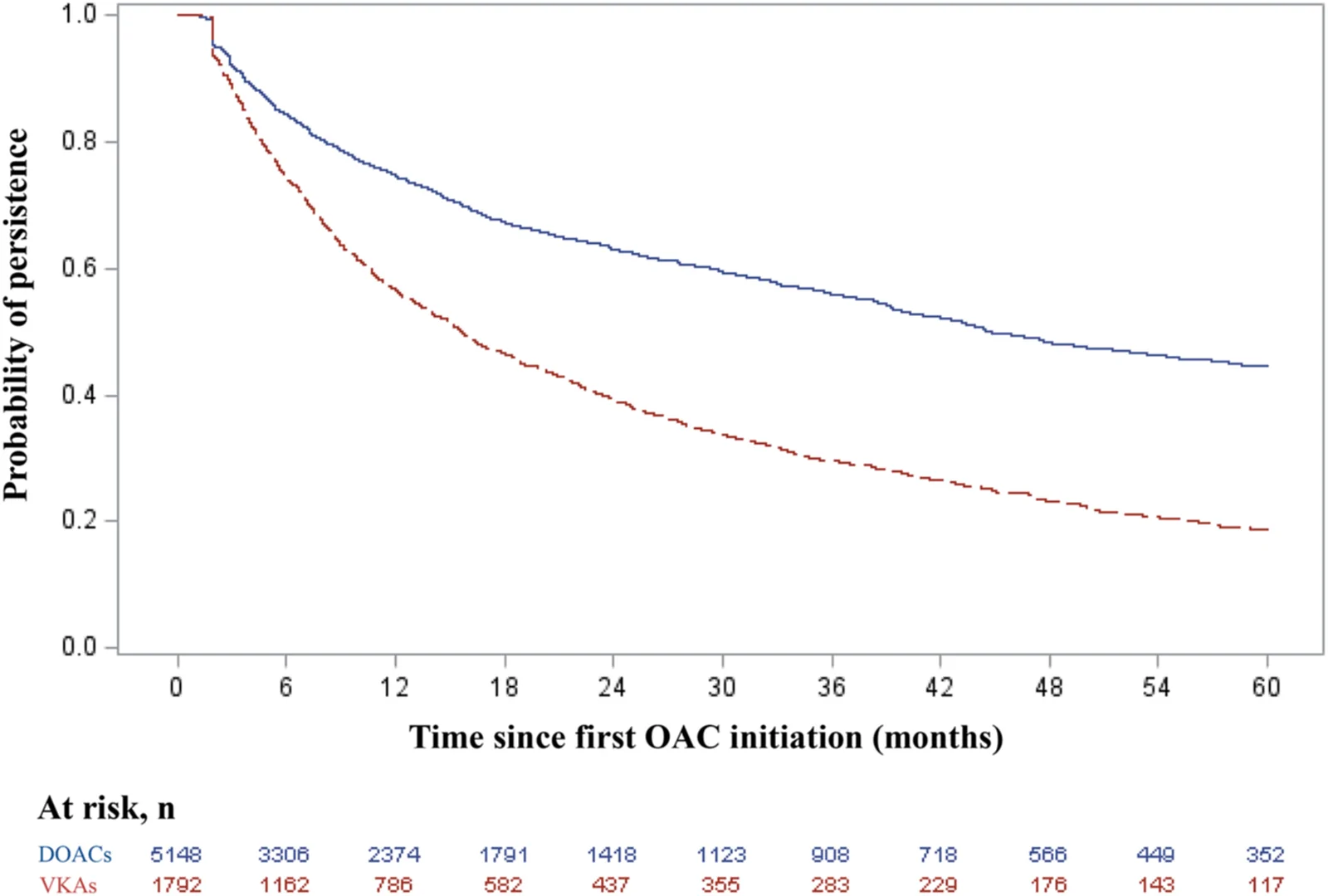
5-Year Persistence With DOACs vs VKAs After First OAC Prescription in Patients With Atrial Fibrillation and Active Cancer in the UK Clinical Practice Research Datalink, 2010 to 2023 OAC = oral anticoagulant; other abbreviations as in Figure 1.

In the overall cohort, OAC-prescribing trends were similar in patients with remote and active cancer, but incidence rates were lower in patients with active cancer (Figure 6). Other analyses showed broadly consistent prescribing patterns between the overall and the active cancer cohorts, with lower rates observed in the latter cohort (Supplemental Tables 4 to 7, Supplemental Figures 2 to 5 and 7 to 9). Finally, results were similar in sensitivity analyses using an alternative active cancer definition (Supplemental Figure 10); grace periods of 15, 60, and 90 days (data not shown); and competing risk analysis (DOACs vs VKAs; subdistribution HR: 0.51; 95% CI: 0.47-0.55).

**Figure 6:**
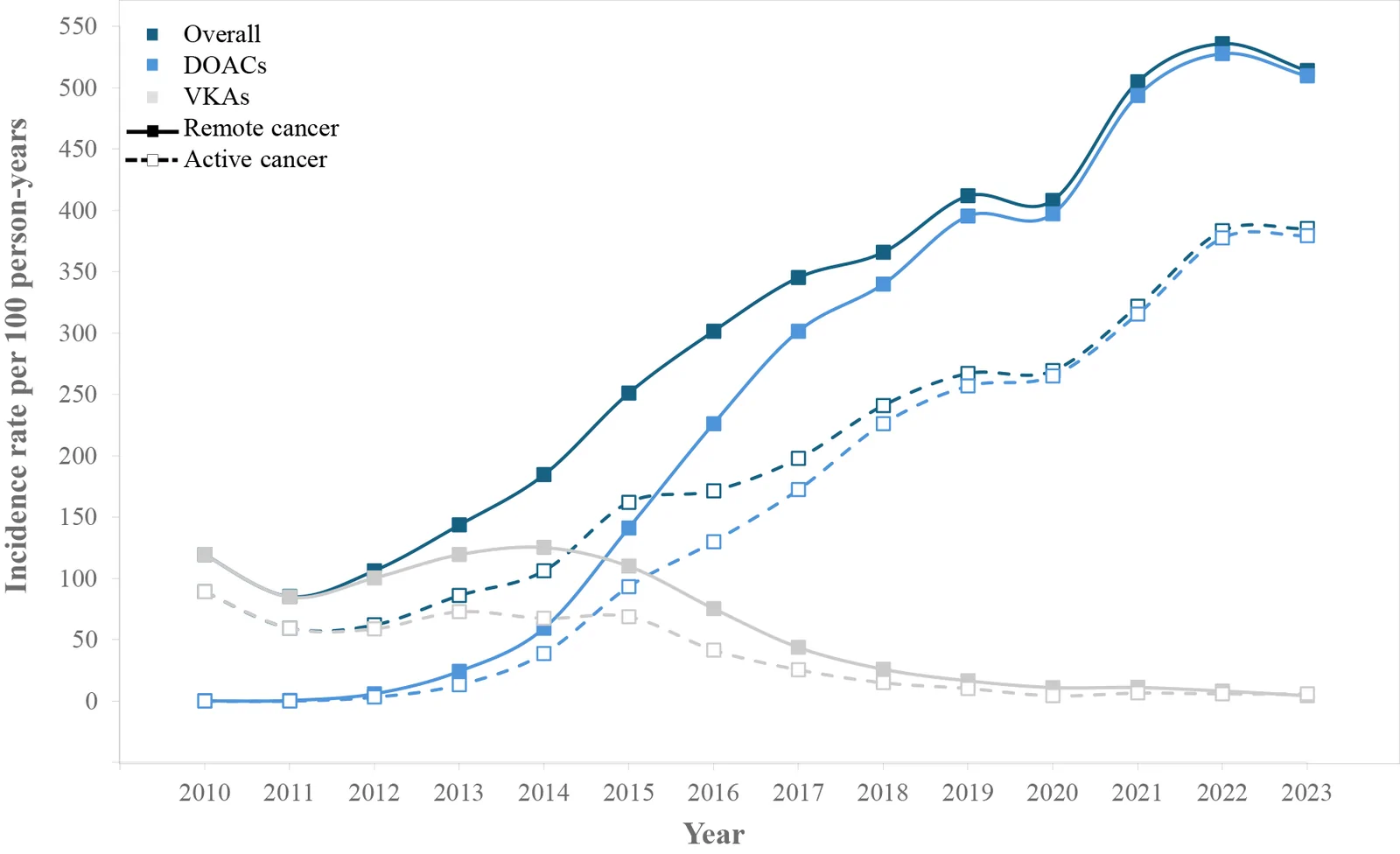
Annual Incidence Rates of Oral Anticoagulant Initiation in Patients With Atrial Fibrillation and Cancer in the UK Clinical Practice Research Datalink, 2010 to 2023, Stratified by Cancer Status (Active vs Remote) Abbreviations as in Figure 1.

## Discussion

In this large population-based cohort study in UK primary care, OAC initiation rates among eligible patients with AF and active cancer increased fourfold between 2010 and 2023, with a shift from VKAs to DOACs. In 2023, apixaban and edoxaban accounted for the vast majority of OAC prescriptions in this population (Central Illustration). However, OAC initiation rates were lower in patients with active cancer than in those with remote cancer. Patients with lung cancer, metastatic cancer, anemia, or dementia, as well as those receiving chemotherapy, were less likely to initiate OACs. Treatment persistence was higher among patients who initiated DOACs than among those who initiated VKAs.

**Central Illustration:**
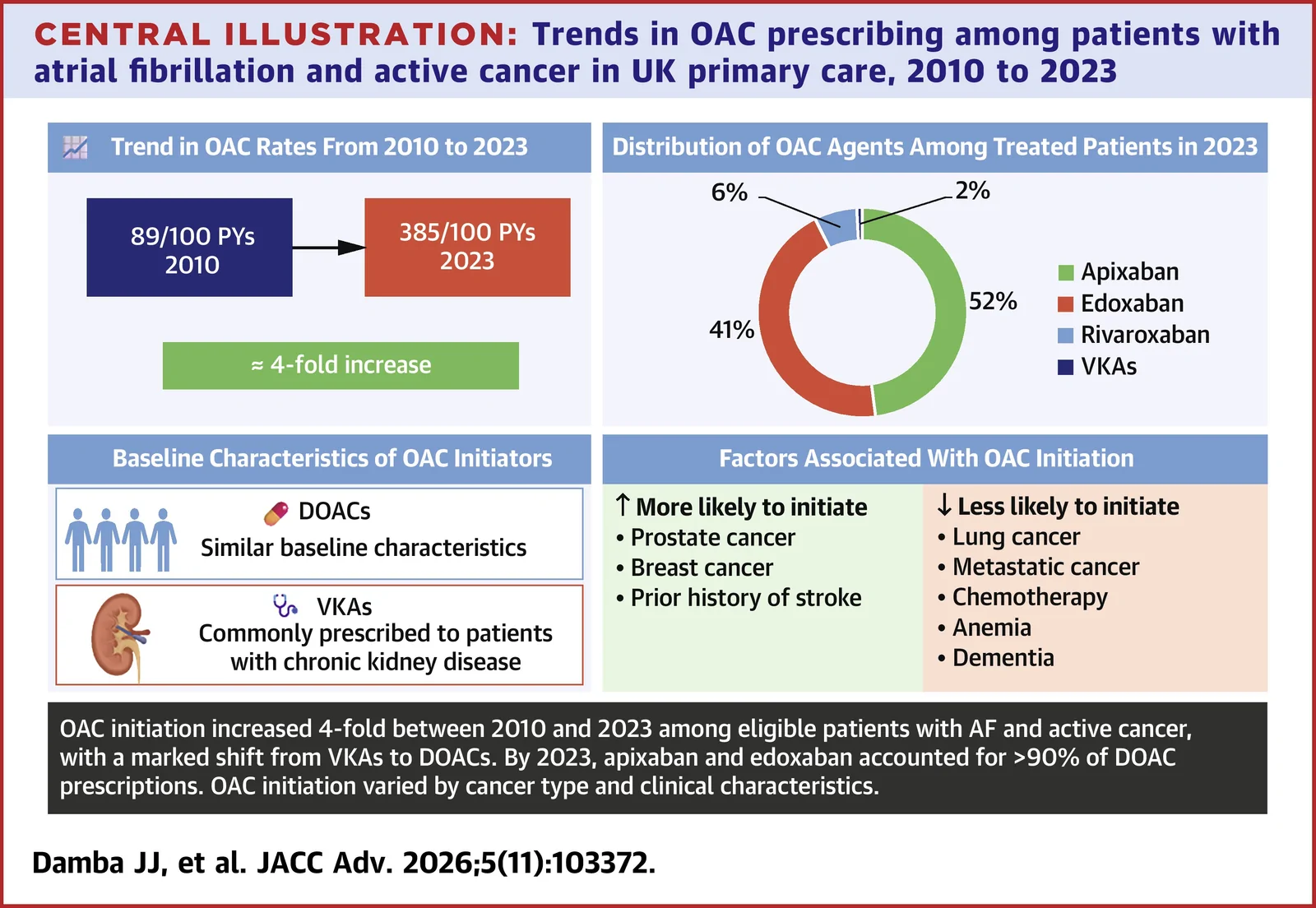
Trends in OAC prescribing among patients with atrial fibrillation and active cancer in UK primary care, 2010 to 2023 DOAC = direct oral anticoagulant; OAC = oral anticoagulant; PY = person-year; VKA = vitamin K antagonist.

Evidence on OAC prescribing among patients with AF and active cancer remains limited. One study conducted between 2011 and 2015 found that approximately 60% of patients with AF and active cancer did not initiate OAC.^33^ Similar findings were reported during the same period in studies of patients with a history of cancer.^16^, ^17^ However, these studies preceded the widespread adoption of DOACs, which are safer and more convenient than VKAs.^9^, ^10^, ^11^, ^12^ Major AF guidelines recommended DOACs as suitable alternatives to VKAs only after 2013,^34^, ^35^, ^36^ following randomized controlled trials that showed comparable efficacy and improved safety.^9^, ^10^, ^11^, ^12^ Subsequent guideline updates published after 2020 established DOACs as the first-line therapy for stroke prevention in eligible patients.^13^, ^14^, ^15^ A UK study spanning 2009 to 2019 had already reported an upward trend, with approximately 67% of patients with AF and active cancer prescribed OACs.^31^ Consistent with this trend, we observed a marked increase in OAC initiation rates over time, and DOACs were the most prescribed OACs in 2023.

Apixaban was the most commonly prescribed OAC across all four UK nations in recent years, followed by edoxaban in England and Scotland. By 2023, apixaban and edoxaban had relatively similar initiation rates in these 2 nations. The high adoption of edoxaban may reflect national guidance promoting its use in England and Scotland. Indeed, National Health Service (NHS) Scotland and NHS England encouraged practices to initiate treatment with edoxaban on cost-effectiveness grounds in 2018 and 2021, respectively,^37^, ^38^, ^39^, ^40^ and in 2022, NHS England added financial incentives to support this initiative.^41^ In Wales and Northern Ireland, where, to our knowledge, no such policy was implemented, edoxaban-prescribing rates remained substantially lower than apixaban-prescribing rates.

Major AF guidelines do not provide specific recommendations on the choice of DOACs.^13^, ^14^, ^15^ In practice, clinicians may weigh pharmacokinetics, pharmacodynamics, clinical evidence on safety and effectiveness, and cost when deciding which DOAC to prescribe. Head-to-head DOAC comparisons from observational studies suggest that apixaban has a more favorable bleeding profile than rivaroxaban and dabigatran, likely contributing to its preferential use among patients with AF and active cancer.^21^, ^32^ Edoxaban use is convenient, with once-daily dosing and no food requirements,^42^ but comparative safety and effectiveness studies with other DOACs in this population remain limited.^21^, ^32^ Uptake of rivaroxaban and dabigatran has been hampered by concerns about the risk of bleeding, which may explain their lower use reported in many studies.^17^, ^21^, ^32^, ^39^ Finally, although the use of VKAs declined significantly over the study period, they remain commonly used in some patients, particularly those with chronic kidney disease. Indeed, in our study, 31.4% of patients prescribed VKAs had chronic kidney disease, compared with 22.9% to 26% across DOAC groups. This is expected, as all DOACs undergo varying degrees of renal elimination (apixaban 27%, rivaroxaban 36%, edoxaban 50%, and dabigatran 80%), raising concerns about drug accumulation and an increased risk of bleeding in patients with kidney impairment.^42^

Several patient characteristics may also influence anticoagulation decisions in this high-risk population. In our study, age was not associated with OAC initiation among patients eligible for oral anticoagulation. This contrasts with 2 U.S. cohorts, which reported higher OAC initiation in older patients in broader cancer populations not restricted to patients with active cancer or those meeting guideline-based eligibility criteria.^16^, ^17^ Indeed, stroke risk rises sharply with age, approximately doubling with each decade after 55 years,^43^ which may explain the higher OAC initiation observed among older patients in these broader populations. However, among patients already eligible for anticoagulation, cancer-related characteristics may have a greater influence on prescribing decisions than age. We found that patients with solid cancers such as prostate and breast cancer were more likely to initiate OACs, whereas those with lung or metastatic cancers were less likely to do so, consistent with the findings from 2 U.S. cohorts.^16^, ^17^ Similarly, active chemotherapy was associated with lower OAC initiation. Indeed, patients with breast cancer have an elevated risk of stroke without a comparable increase in the risk of major bleeding.^7^ By contrast, those with lung cancer may have a higher bleeding risk owing to tumor necrosis, exfoliation of the tumor surface, or erosion of cancerous tissue into adjacent blood vessels.^7^, ^44^ Chemotherapy raises bleeding concerns through treatment-induced thrombocytopenia, drug-drug interactions, and mucosal injury (eg, mucositis or gastrointestinal damage).^45^

Patients with active cancer also have a higher burden of comorbidities,^46^ some of which may further influence OAC-prescribing decisions. For instance, anemia and dementia were associated with lower OAC initiation. Dementia may complicate OAC prescribing because of cognitive impairment, increased fall risk, and limited life expectancy. Aspirin prescribing was common among patients with AF and active cancer who did not initiate OACs. A previous UK study of patients with AF and cancer also reported frequent aspirin-only prescribing between 2009 and 2013, particularly among patients with HAS-BLED scores ≥3.^31^ Although aspirin is not recommended for stroke prevention in AF,^13^, ^14^, ^15^ it may have been prescribed in settings where OAC was withheld because of additional comorbidities associated with a higher perceived bleeding risk. In some patients, aspirin may also have been prescribed for secondary prevention after an ischemic event, as patients with active cancer and a history of ischemic stroke or other arterial thromboembolic events are at high risk of recurrent vascular events, particularly during the year after cancer diagnosis.^47^, ^48^, ^49^

Beyond OAC initiation, we also found that OAC persistence was higher among patients with AF and active cancer initiating DOACs than among those initiating VKAs. DOACs offer practical advantages, including fixed dosing, fewer monitoring requirements, and a lower potential for drug-drug interactions with cancer treatments, all of which may contribute to the higher persistence observed in our study.^42^ Moreover, the elevated bleeding risk inherent in active cancer, together with the higher bleeding risk with VKAs than with DOACs, may have contributed to more treatment discontinuations and lower persistence with VKAs.

### Study limitations

This study provides the most recent assessment of OAC initiation in patients with AF and active cancer in the United Kingdom. Our extended study period allows for the examination of long-term trends, including the period following the introduction of edoxaban. We report clinical characteristics and factors associated with OAC initiation in recent years. We also report treatment persistence with DOACs compared with VKAs, an aspect not assessed in prior studies conducted in this population.

Nonetheless, this study has several limitations. First, the active-cancer cohort, defined according to the recency of the first recorded cancer diagnosis, may have been heterogeneous with respect to cancer status. Indeed, some patients classified as having active cancer may have completed treatment or no longer had a clinically active disease by the time of AF diagnosis. However, the sensitivity analysis using a more stringent definition yielded similar results. Second, information on several predictors of OAC initiation in patients with active cancer, such as tumor grade, cancer stage, tumor size, palliative care, performance status, life expectancy, and severity of thrombocytopenia, was unavailable or incompletely captured. We used the presence of metastasis as a proxy for advanced cancer stage, although this variable is not consistently recorded in CPRD. As such, OAC-prescribing trends among patients with advanced or metastatic cancer may not have been fully characterized. More broadly, OAC noninitiation should be interpreted cautiously because the available data did not permit distinguishing between clinically appropriate withholding and potential underprescribing. Third, the CPRD captures prescriptions issued in primary care only, which may underestimate the rates of OAC initiation in this population. However, GPs manage most OAC prescriptions for patients with AF in the United Kingdom. Moreover, our findings remain informative for prescribing trends in UK primary care.

## Conclusions

OAC initiation among eligible patients with AF and active cancer has increased in recent years, with DOACs largely replacing VKAs. Apixaban and edoxaban are now the most commonly prescribed agents. Nonetheless, a substantial proportion of eligible patients did not initiate an OAC during the study period, with lower initiation among those with lung cancer, metastatic cancer, dementia, or anemia. Given the rapid uptake of edoxaban, further comparative studies are needed to clarify its effectiveness and safety in this high-risk population.

Perspectives **COMPETENCY IN CLINICAL KNOWLEDGE:** This study highlights a substantial increase in OAC initiation among patients with AF and active cancer in the last decade, with a shift toward DOACs. It also identifies clinical characteristics and comorbidities associated with OAC initiation. These prescribing trends may help clinicians address potential gaps in anticoagulation use and optimize stroke-prevention strategies in patients with AF and active cancer.

**TRANSLATIONAL OUTLOOK:** Given the high uptake of edoxaban, future studies are warranted to assess its comparative safety and effectiveness relative to other DOACs in patients with AF and active cancer. Additional research should also explore the comparative safety of different OACs in subgroups less likely to initiate treatment, including patients with lung cancer, metastatic cancer, or dementia.

## Funding support and author disclosures

Dr Damba is the recipient of a Vanier Canada Graduate Scholarship from the Canadian Institutes of Health Research. Dr Azoulay is a recipient of a Chercheur-Boursier Salary Award from the Fonds de recherche du Québec-Santé. All other authors have reported that they have no relationships relevant to the contents of this paper to disclose.

## References

[1] Wolf P.A., Abbott R.D., Kannel W.B. (1991). Atrial fibrillation as an independent risk factor for stroke: the framingham study. Stroke.

[2] Khorana A.A., Noble S., Lee A.Y.Y. (2018). Role of direct oral anticoagulants in the treatment of cancer-associated venous thromboembolism: guidance from the SSC of the ISTH. J Thromb Haemost.

[3] Suzuki Y., Kaneko H., Okada A. (2024). Risk of cancer history in cardiovascular disease among individuals with hypertension. Hypertension Research.

[4] Yun J.P., Choi E.K., Han K.D. (2021). Risk of atrial fibrillation according to cancer type: a nationwide population-based study. JACC CardioOncol.

[5] Conen D., Wong J.A., Sandhu R.K. (2016). Risk of malignant cancer among women with new-onset atrial fibrillation. JAMA Cardiol.

[6] Rahman F., Ko D., Benjamin E.J. (2016). Association of atrial fibrillation and cancer. JAMA Cardiol.

[7] Ajabnoor A.M., Parisi R., Zghebi S.S. (2023). Common cancer types and risk of stroke and bleeding in patients with nonvalvular atrial fibrillation: a population-based study in England. J Am Heart Assoc.

[8] Aspberg S., Yu L., Gigante B., Smedby K.E., Singer D.E. (2020). Risk of ischemic stroke and major bleeding in patients with atrial fibrillation and cancer. J Stroke Cerebrovasc Dis.

[9] Connolly S.J., Ezekowitz M.D., Yusuf S. (2009). Dabigatran versus warfarin in patients with atrial fibrillation. N Engl J Med.

[10] Patel M.R., Mahaffey K.W., Garg J. (2011). Rivaroxaban versus warfarin in nonvalvular atrial fibrillation. N Engl J Med.

[11] Granger C.B., Alexander J.H., McMurray J.J. (2011). Apixaban versus warfarin in patients with atrial fibrillation. N Engl J Med.

[12] Giugliano R.P., Ruff C.T., Braunwald E. (2013). Edoxaban versus warfarin in patients with atrial fibrillation. N Engl J Med.

[13] Jones N.R., Round T., Rajappan K. (2022). Atrial fibrillation: NICE 2021 update and the focus on anticoagulation. Br J Gen Pract.

[14] Van Gelder I.C., Rienstra M., Bunting K.V. (2024). 2024 ESC guidelines for the management of atrial fibrillation developed in collaboration with the European Association for Cardio-Thoracic Surgery (EACTS): developed by the task force for the management of atrial fibrillation of the European Society of Cardiology (ESC), with the special contribution of the European Heart Rhythm Association (EHRA) of the ESC. Endorsed by the European Stroke Organisation (ESO). Eur Heart J.

[15] Andrade J.G., Aguilar M., Atzema C. (2020). The 2020 Canadian Cardiovascular Society/Canadian Heart Rhythm Society Comprehensive guidelines for the management of atrial fibrillation. Can J Cardiol.

[16] Fradley M.G., Ellenberg K., Alomar M. (2020). Patterns of anticoagulation use in patients with cancer with atrial fibrillation and/or atrial flutter. JACC CardioOncol.

[17] Ardeshirrouhanifard S., An H., Goyal R.K. (2022). Use of oral anticoagulants among individuals with cancer and atrial fibrillation in the United States, 2010-2016. Pharmacotherapy.

[18] European Pharmaceutical Review (2015). Daiichi Sankyo’s edoxaban launched in the UK. https://www.europeanpharmaceuticalreview.com/news/33248/daiichi-sankyos-edoxaban-launched-in-the-uk/.

[19] Lau W.C.Y., Torre C.O., Man K.K.C. (2022). Comparative effectiveness and safety between Apixaban, Dabigatran, Edoxaban, and rivaroxaban among patients with atrial fibrillation : a multinational population-based cohort study. Ann Intern Med.

[20] Chiv R., Beradid S., Suissa S., Renoux C. (2024). Effectiveness and safety of edoxaban compared with Apixaban in elderly patients with nonvalvular atrial fibrillation: a real-world population-based cohort study. Stroke.

[21] Deitelzweig S., Keshishian A.V., Zhang Y. (2021). Effectiveness and safety of oral anticoagulants among nonvalvular atrial fibrillation patients with active cancer. JACC CardioOncol.

[22] Loo S.Y., Dell'Aniello S., Huiart L., Renoux C. (2017). Trends in the prescription of novel oral anticoagulants in UK primary care. Br J Clin Pharmacol.

[23] Herrett E., Gallagher A.M., Bhaskaran K. (2015). Data resource profile: Clinical Practice Research Datalink (CPRD). Int J Epidemiol.

[24] Wolf A., Dedman D., Campbell J. (2019). Data resource profile: Clinical Practice Research Datalink (CPRD) Aurum. Int J Epidemiol.

[25] Clinical Practice Research Datalink (2025). CPRD Data Access. https://www.cprd.com/data.

[26] Herrett E., Thomas S.L., Schoonen W.M., Smeeth L., Hall A.J. (2010). Validation and validity of diagnoses in the General Practice Research Database: a systematic review. Br J Clin Pharmacol.

[27] Hagberg K.W., Vasilakis-Scaramozza C., Persson R. (2022). Quality and completeness of malignant cancer recording in United Kingdom Clinical Practice Research Datalink Aurum compared to hospital episode statistics. Ann Cancer Epidemiol.

[28] Pharmalife (2011). Xarelto gains new licences in UK. https://pharmafile.com/news/xarelto-gains-new-licences-uk/.

[29] Lip G.Y., Nieuwlaat R., Pisters R., Lane D.A., Crijns H.J. (2010). Refining clinical risk stratification for predicting stroke and thromboembolism in atrial fibrillation using a novel risk factor-based approach: the euro heart survey on atrial fibrillation. Chest.

[30] Pisters R., Lane D.A., Nieuwlaat R., de Vos C.B., Crijns H.J., Lip G.Y. (2010). A novel user-friendly score (HAS-BLED) to assess 1-year risk of major bleeding in patients with atrial fibrillation: the Euro Heart Survey. Chest.

[31] Ajabnoor A.M., Parisi R., Zghebi S.S. (2024). Oral anticoagulant prescribing among patients with cancer and atrial fibrillation in England, 2009-2019. Cancer.

[32] Shah S., Norby F.L., Datta Y.H. (2018). Comparative effectiveness of direct oral anticoagulants and warfarin in patients with cancer and atrial fibrillation. Blood Adv.

[33] Malavasi V.L., Fantecchi E., Gianolio L. (2019). Atrial fibrillation in patients with active malignancy and use of anticoagulants: Under-prescription but no adverse impact on all-cause mortality. Eur J Intern Med.

[34] National Clinical Guideline C. National Institute for Health and Clinical Excellence: Guidance (2014).

[35] Meschia J.F., Bushnell C., Boden-Albala B. (2014). Guidelines for the primary prevention of stroke. Stroke.

[36] Kirchhof P., Benussi S., Kotecha D. (2016). 2016 ESC guidelines for the management of atrial fibrillation developed in collaboration with EACTS. Eur H J.

[37] NHS England Thousands of lives saved by the NHS thanks to rapid rollout of blood thinners 2023. https://www.england.nhs.uk/2023/11/thousands-of-lives-saved-by-the-nhs-thanks-to-rapid-rollout-of-blood-thinners/.

[38] NHS Scotland (2018). Edoxaban switch programme—frequently asked questions. https://www.nhstaysideadtc.scot.nhs.uk/netFormulary/PDF/Edoxaban%20Switch%20FAQ.pdf.

[39] Ho K.H., van Hove M., Leng G. (2020). Trends in anticoagulant prescribing: a review of local policies in English primary care. BMC Health Services Research.

[40] Mahase E. (2023). NHS England drive sees almost half a million people started on anticoagulant drugs. Bmj.

[41] The pharmaceutical journal (2022). Edoxaban switching scheme: worth the risks?. https://pharmaceutical-journal.com/article/feature/edoxaban-switching-scheme-worth-the-risks.

[42] Katzung B.G., Vanderah T.W. (2021).

[43] Yousufuddin M., Young N. (2019). Aging and ischemic stroke. Aging (Albany NY).

[44] Gershman E., Guthrie R., Swiatek K., Shojaee S. (2019). Management of hemoptysis in patients with lung cancer. Ann Transl Med.

[45] Johnstone C., Rich S.E. (2017). Bleeding in cancer patients and its treatment: a review. Annals of Palliative Medicine.

[46] Davis M.K., Lim H., Lee A.Y.Y. (2021). Direct oral anticoagulants in patients with cancer and nonvalvular atrial fibrillation. JACC CardioOncol.

[47] Navi B.B., Reiner A.S., Kamel H. (2015). Association between incident cancer and subsequent stroke. Ann Neurol.

[48] Navi B.B., Reiner A.S., Kamel H. (2017). Risk of arterial thromboembolism in patients with cancer. J Am Coll Cardiol.

[49] Navi B.B., Kasner S.E., Cushman M. (2026). Classification and management of ischemic stroke in patients with active cancer: a scientific statement from the American Heart Association. Stroke.

