# Supplementary material for: Trends in Oral Anticoagulant Prescribing for Atrial Fibrillation and Active Cancer in UK Primary Care
Source: JACC Adv. 2026 Oct 23;5(11):103372. doi: 10.1016/j.jacadv.2026.103372 (PMC13721092; doi:10.1016/j.jacadv.2026.103372)
Supplement: Supplementary file 1 [file mmc1.docx]

**Supplemental Appendix**

**Trends in Oral Anticoagulant Prescribing for Atrial Fibrillation and active Cancer in UK Primary Care**

Joseph Junior Damba, MD, MSc^a,b^, Sarah Beradid, MSc^a^, Laurent Azoulay, PhD^a,b,c^, James M. Brophy, MD, PhD^d^, Christel Renoux, MD, PhD^a,b,e,f^

**Contents**

[**Table S1**. Description of clinical characteristics measured at OAC initiation among patients with AF and active cancer in the UK CPRD, 2015–2023.	3](#_Toc236313061)

[**Table S2**. Annual incidence rates of OAC initiation in the year following AF diagnosis among patients with active cancer in the UK CPRD, 2010–2023, with crude and adjusted rate ratios.	4](#_Toc236313062)

[**Table S3**. Annual incidence rates of OAC initiation in the year following AF diagnosis among patients with active cancer in the UK CPRD, 2010–2023, with crude and adjusted rate differences.	5](#_Toc236313063)

[**Table S4**. Annual incidence rates of OAC initiation in the year following AF diagnosis among patients with cancer in the UK CPRD, 2010–2023, with crude and adjusted rate ratios.	6](#_Toc236313064)

[**Table S5**. Annual incidence rates of OAC initiation in the year following AF diagnosis among patients with cancer in the UK CPRD, 2010–2023, with crude and adjusted rate differences.	7](#_Toc236313065)

[**Table S6**. Clinical characteristics of patients with AF and cancer who initiated OACs in the UK CPRD, 2015–2023.	8](#_Toc236313066)

[**Table S7**. Factors associated with OAC initiation in the year following AF diagnosis in patients with cancer in the UK CPRD, 2015–2023.	11](#_Toc236313067)

[**Figure S1**. Flowchart of cohort formation of patients with atrial fibrillation (AF) and cancer in UK Clinical Practice Research Datalink between January 1, 2021, and December 31, 2023...13](#_Toc236313818)

[**Figure S2**. Annual incidence rates of oral anticoagulant initiation in patients with atrial fibrillation and cancer in the UK Clinical Practice Research Datalink, 2010–2023, stratified by sex. (A): active cancer; (B): overall cohort.	14](#_Toc236313819)

[**Figure S3**. Annual incidence rates of oral anticoagulant initiation in patients with atrial fibrillation and cancer in the UK Clinical Practice Research Datalink, 2010–2023, stratified by HAS–BLED score. (A) active cancer; (B) overall cohort.	15](#_Toc236313820)

[**Figure S4**. Annual incidence rates of oral anticoagulant initiation in patients with atrial fibrillation and cancer in the UK Clinical Practice Research Datalink, 2010–2023, stratified by bleeding history. (A) active cancer; (B) overall cohort.	16](#_Toc236313821)

[**Figure S5**. Annual incidence rates of oral anticoagulant initiation in patients with atrial fibrillation and active cancer in the UK Clinical Practice Research Datalink, 2010–2023, stratified by UK nations.	17](#_Toc236313822)

[**Figure S6**. Annual incidence rates of oral anticoagulant initiation in patients with atrial fibrillation and cancer (overall cohort) in the UK Clinical Practice Research Datalink, 2010–2023, stratified by UK nations.	18](#_Toc236313823)

[**Figure S7**. Annual incidence rates of oral anticoagulant initiation in patients with atrial fibrillation and cancer (overall cohort) in the UK Clinical Practice Research Datalink, 2010–2023, by individual direct oral anticoagulants (DOACs) and vitamin K antagonists (VKAs).	19](#_Toc236313824)

[**Figure S8**. Annual incidence rates of oral anticoagulant initiation in patients with atrial fibrillation and cancer (overall cohort) in the UK Clinical Practice Research Datalink, 2010–2023, stratified by age. (A) DOACs; (B) VKAs.	20](#_Toc236313825)

[**Figure S9**. Five-year persistence with DOACs versus VKAs after first OAC initiation in patients with atrial fibrillation and cancer (overall cohort) in the UK Clinical Practice Research Datalink, 2010–2023.	21](#_Toc236313826)

[**Figure S10**. Annual incidence rates of oral anticoagulant initiation in patients with atrial fibrillation and active cancer in the UK Clinical Practice Research Datalink, 2010–2023, overall and by direct oral anticoagulants (DOACs) vs vitamin K antagonists (VKAs). (A) cancer diagnosed within 1 year before AF diagnosis; (B) cancer diagnosed within 6 months before AF diagnosis, receipt of radiotherapy or chemotherapy, cancer-directed surgery, or a recorded metastatic disease codes in 6 months before AF diagnosis.	22](#_Toc236313827)

**Table S1**. Description of clinical characteristics measured at OAC initiation among patients with AF and active cancer in the UK CPRD, 2015–2023.

| **Patient demographics** | Age and sex |
| --- | --- |
| **Lifestyle risk factors** | Risk factors included body mass index and smoking status, measured within five years before OAC initiation. |
| **Cancer-related variables** | Presence of metastases, and cancer-related treatments such as radiotherapy, chemotherapy and surgery, all measured in the year before OAC initiation. |
| **Comorbidities** | Comorbidities were measured at anytime before OAC initiation, including hypertension, congestive heart failure, myocardial infarction, coronary artery disease, coronary artery bypass surgery or percutaneous coronary intervention, prior ischemic stroke/transient ischemic attack, systemic embolism, peripheral artery disease, prior bleeding, thrombocytopenia; anemia, depression, dementia or mild cognitive impairment, Parkinson’s disease, history of falls, chronic obstructive pulmonary disease, acute kidney injury, chronic kidney disease, liver disease, and diabetes mellitus. |
| **Medications** | Medications were measured in the year before OAC initiation, including antidiabetic medications (metformin, sulfonylureas, glucagon-like peptide 1 receptor agonists, dipeptidyl peptidase 4 inhibitors, sodium-glucose cotransporter-2, insulin, others), antihypertensive drugs (beta-blockers, thiazides, other diuretics, angiotensin-converting enzyme inhibitors, angiotensin II receptor blockers, calcium channel blockers), antiarrhythmics, digoxin, antiplatelet agents, lipid-lowering drugs, non-steroidal anti-inflammatory drugs, opioids, systemic corticosteroids, antidepressants, antipsychotics, benzodiazepines and hypnotics, antiepileptic drugs, proton pump inhibitors, H2 blockers, and hormone replacement therapy. |
| **OAC dose at initiation** | Low-dose regimens were defined as dabigatran 110 mg twice daily, rivaroxaban 15 mg once daily, apixaban 2.5 mg twice daily, and edoxaban 30 mg once daily. Standard-dose regimens were defined as dabigatran 150 mg twice daily, rivaroxaban 20 mg once daily, apixaban 5 mg twice daily, and edoxaban 60 mg once daily. |

Abbreviations: AF = atrial fibrillation; CPRD = Clinical Practice Research Datalink; OAC = oral anticoagulant; UK = United Kingdom.

# **Table S2**. Annual incidence rates of OAC initiation in the year following AF diagnosis among patients with active cancer in the UK CPRD, 2010–2023, with crude and adjusted rate ratios.

| **Year** | **Incidence rate^a^**  **(95% CI)** | **Crude RR vs 2010** | **Adjusted^b^ RR vs 2010**  **(95% CI)** | **Adjusted^b^ RR vs previous year**  **(95% CI)** |
| --- | --- | --- | --- | --- |
| 2010 | 89.1 (73.7–107.7) | **Reference** | **1.00** (Ref) | **1.00** (Ref for 2011 only) |
| 2011 | 59.3 (49.4–71.3) | 0.67 | 0.67 (0.55–0.81) | 0.67 (0.55–0.81) |
| 2012 | 61.9 (51.7–74.3) | 0.70 | 0.70 (0.58–0.85) | 1.05 (0.86–1.27) |
| 2013 | 85.9 (73.3–100.8) | 0.96 | 0.97 (0.82–1.16) | 1.39 (1.17–1.65) |
| 2014 | 106.1 (91.5–122.9) | 1.19 | 1.19 (1.01–1.41) | 1.23 (1.04–1.44) |
| 2015 | 161.9 (142.6–183.9) | 1.82 | 1.83 (1.58–2.12) | 1.53 (1.32–1.77) |
| 2016 | 171.4 (151.1–194.5) | 1.92 | 1.93 (1.67–2.23) | 1.06 (0.92–1.22) |
| 2017 | 197.7 (175.2–223.0) | 2.22 | 2.23 (1.94–2.56) | 1.15 (1.00–1.33) |
| 2018 | 240.9 (214.8–270.1) | 2.70 | 2.72 (2.38–3.11) | 1.22 (1.07–1.40) |
| 2019 | 267.0 (238.2–299.3) | 3.00 | 3.00 (2.62–3.43) | 1.10 (0.96–1.26) |
| 2020 | 269.2 (238.3–303.9) | 3.02 | 3.02 (2.62–3.47) | 1.01 (0.87–1.16) |
| 2021 | 321.8 (285.5–362.7) | 3.61 | 3.61 (3.14–4.15) | 1.20 (1.04–1.38) |
| 2022 | 383.3 (342.2–429.3) | 4.30 | 4.27 (3.74–4.89) | 1.18 (1.03–1.35) |
| 2023 | 385.0 (344.3–430.6) | 4.32 | 4.32 (3.78–4.93) | 1.01 (0.88–1.15) |

^a^Crude incidence rate per 100 person-years.

^b^Adjusted for age and sex.

Abbreviations: AF = atrial fibrillation; CI = confidence interval; CPRD = Clinical Practice Research Datalink; OAC = oral anticoagulant; RR = rate ratio; UK = United Kingdom.

# **Table S3**. Annual incidence rates of OAC initiation in the year following AF diagnosis among patients with active cancer in the UK CPRD, 2010–2023, with crude and adjusted rate differences.

| **Year** | **Incidence rate^a^**  **(95% CI)** | **Crude RD vs 2010** | **Adjusted^b^ RD vs 2010**  **(95% CI)** | **Adjusted^b^ RD vs previous year**  **(95% CI)** |
| --- | --- | --- | --- | --- |
| 2010 | 89.1 (73.7–107.7) | **Reference** | **1.00** (Ref) | **1.00** (Ref for 2011 only) |
| 2011 | 59.3 (49.4–71.3) | -29.73 | -27.84 (-49.96–-9.51) | -27.84 (-48.33–-7.35) |
| 2012 | 61.9 (51.7–74.3) | -27.14 | -25.18 (-47.55–-6.73) | 2.66 (-13.42–18.73) |
| 2013 | 85.9 (73.3–100.8) | -3.15 | -2.27 (-25.03–18.72) | 22.92 (4.45–41.38) |
| 2014 | 106.1 (91.5–122.9) | 16.98 | 16.27 (-6.17–40.13) | 18.54 (-3.38–40.46) |
| 2015 | 161.9 (142.6–183.9) | 72.83 | 69.61 (46.07–99.59) | 53.34 (25.34–81.34) |
| 2016 | 171.4 (151.1–194.5) | 82.31 | 78.41 (54.75–109.87) | 8.80 (-23.76–41.36) |
| 2017 | 197.7 (175.2–223.0) | 108.58 | 103.48 (79.23–137.92) | 25.07 (-10.13–60.27) |
| 2018 | 240.9 (214.8–270.1) | 151.79 | 145.13 (119.34–184.23) | 41.65 (1.26–82.04) |
| 2019 | 267.0 (238.2–299.3) | 177.92 | 168.27 (142.98–212.85) | 23.14 (-22.60–68.88) |
| 2020 | 269.2 (238.3–303.9) | 180.07 | 169.65 (143.13–217.02) | 1.38 (-47.99–50.74) |
| 2021 | 321.8 (285.5–362.7) | 232.72 | 219.82 (190.56–274.88) | 50.18 (-5.35–105.70) |
| 2022 | 383.3 (342.2–429.3) | 294.18 | 275.54 (247.43–340.92) | 55.72 (-8.72–120.15) |
| 2023 | 385.0 (344.3–430.6) | 295.95 | 279.11 (249.57–342.34) | 3.57 (-65.09–72.24) |

^a^Crude incidence rate per 100 person-years.

^b^Adjusted for age and sex.

Abbreviations: AF = atrial fibrillation; CI = confidence interval; CPRD = Clinical Practice Research Datalink; OAC = oral anticoagulant; RD = rate difference; UK = United Kingdom.

# **Table S4**. Annual incidence rates of OAC initiation in the year following AF diagnosis among patients with cancer in the UK CPRD, 2010–2023, with crude and adjusted rate ratios.

| **Year** | **Incidence rate^a^**  **(95% CI)** | **Crude RR vs 2010** | **Adjusted^b^ RR vs 2010**  **(95% CI)** | **Adjusted^b^ RR vs previous year**  **(95% CI)** |
| --- | --- | --- | --- | --- |
| 2010 | 114.8 (106.8–123.3) | **Reference** | **1.00** (Ref) | **1.00** (Ref for 2011 only) |
| 2011 | 81.7 (76.7–87.0) | 0.71 | 0.71 (0.67–0.76) | 0.71 (0.67–0.76) |
| 2012 | 100.3 (94.7–106.2) | 0.87 | 0.88 (0.82–0.93) | 1.23 (1.15–1.31) |
| 2013 | 135.6 (128.7–142.8) | 1.18 | 1.18 (1.12–1.25) | 1.35 (1.27–1.43) |
| 2014 | 173.0 (164.7–181.6) | 1.51 | 1.51 (1.43–1.60) | 1.28 (1.21–1.35) |
| 2015 | 238.0 (227.8–248.7) | 2.07 | 2.07 (1.97–2.18) | 1.37 (1.30–1.44) |
| 2016 | 281.4 (269.6–293.7) | 2.45 | 2.45 (2.33–2.58) | 1.18 (1.13–1.24) |
| 2017 | 322.3 (309.4–335.8) | 2.81 | 2.81 (2.68–2.95) | 1.15 (1.09–1.20) |
| 2018 | 347.6 (334.0–361.8) | 3.03 | 3.04 (2.89–3.19) | 1.08 (1.03–1.13) |
| 2019 | 391.0 (376.0–406.7) | 3.41 | 3.42 (3.27–3.59) | 1.13 (1.08–1.18) |
| 2020 | 387.3 (371.0–404.4) | 3.37 | 3.39 (3.22–3.56) | 0.99 (0.94–1.04) |
| 2021 | 479.3 (460.4–499.0) | 4.18 | 4.19 (3.99–4.40) | 1.24 (1.18–1.30) |
| 2022 | 516.2 (496.7–536.5) | 4.50 | 4.51 (4.30–4.72) | 1.08 (1.03–1.13) |
| 2023 | 498.4 (480.1–517.4) | 4.34 | 4.35 (4.16–4.56) | 0.97 (0.92–1.01) |

^a^Crude incidence rate per 100 person-years.

^b^Adjusted for age and sex.

Abbreviations: AF = atrial fibrillation; CI = confidence interval; CPRD = Clinical Practice Research Datalink; OAC = oral anticoagulant; RR = rate ratio; UK = United Kingdom.

# **Table S5**. Annual incidence rates of OAC initiation in the year following AF diagnosis among patients with cancer in the UK CPRD, 2010–2023, with crude and adjusted rate differences.

| **Year** | **Incidence rate^a^**  **(95% CI)** | **Crude RD vs 2010** | **Adjusted^b^ RD vs 2010**  **(95% CI)** | **Adjusted^b^ RD vs previous year**  **(95% CI)** |
| --- | --- | --- | --- | --- |
| 2010 | 114.8 (106.8–123.3) | **Reference** | **1.00** (Ref) | **1.00** (Ref for 2011 only) |
| 2011 | 81.7 (76.7–87.0) | -33.10 | -30.85 (-40.63–-21.07) | -30.85 (-40.63–-21.07) |
| 2012 | 100.3 (94.7–106.2) | -14.47 | -13.19 (-23.36–-3.01) | 17.66 (9.69–25.63) |
| 2013 | 135.6 (128.7–142.8) | 20.80 | 19.70 (8.62–30.78) | 32.88 (23.37–42.40) |
| 2014 | 173.0 (164.7–181.6) | 58.17 | 55.13 (42.92–67.35) | 35.44 (23.76–47.11) |
| 2015 | 238.0 (227.8–248.7) | 123.24 | 115.54 (101.42–129.66) | 60.41 (45.82–75.00) |
| 2016 | 281.4 (269.6–293.7) | 166.60 | 156.37 (140.76–171.97) | 40.82 (23.30–58.35) |
| 2017 | 322.3 (309.4–335.8) | 207.55 | 194.74 (177.90–211.59) | 38.38 (18.59–58.16) |
| 2018 | 347.6 (334.0–361.8) | 232.81 | 219.25 (201.58–236.93) | 24.51 (3.05–45.97) |
| 2019 | 391.0 (376.0–406.7) | 276.22 | 260.94 (241.72–280.15) | 41.68 (18.31–65.05) |
| 2020 | 387.3 (371.0–404.4) | 272.56 | 257.03 (236.83–277.23) | -3.91 (-29.24–21.42) |
| 2021 | 479.3 (460.4–499.0) | 364.54 | 343.46 (320.37–366.55) | 86.43 (58.05–114.81) |
| 2022 | 516.2 (496.7–536.5) | 401.46 | 377.88 (353.80–401.95) | 34.42 (3.16–65.67) |
| 2023 | 498.4 (480.1–517.4) | 383.64 | 361.11 (338.23–383.99) | -16.77 (-47.87–14.34) |

^a^Crude incidence rate per 100 person-years.

^b^Adjusted for age and sex.

Abbreviations: AF = atrial fibrillation; CI = confidence interval; CPRD = Clinical Practice Research Datalink; OAC = oral anticoagulant; RD = rate difference; UK = United Kingdom.

# **Table S6**. Clinical characteristics of patients with AF and cancer who initiated OACs in the UK CPRD, 2015–2023.

|  | **Dabigatran (n=927)** | **Rivaroxaban (n=10,826)** | **Apixaban (n=29,341)** | **Edoxaban (n=9,740)** | **VKAs (n=5,386)** |
| --- | --- | --- | --- | --- | --- |
| **Age, mean (SD)** | 78.7 (7.5) | 78.9 (8.0) | 79.8 (8.0) | 79.7 (7.9) | 78.7 (7.7) |
| **Age categories** |  |  |  |  |  |
| 50–59 | 9 (1.0) | 158 (1.5) | 354 (1.2) | 121 (1.2) | 78 (1.4) |
| 60–69 | 106 (11.4) | 1,186 (11.0) | 2,734 (9.3) | 872 (8.9) | 542 (10.1) |
| 70–79 | 354 (38.2) | 4,202 (38.8) | 10,608 (36.2) | 3,624 (37.2) | 2,146 (39.8) |
| ≥80 | 458 (49.4) | 5,280 (48.8) | 15,645 (53.3) | 5,123 (52.6) | 2,620 (48.6) |
| **Sex** |  |  |  |  |  |
| Male | 527 (56.9) | 5,824 (53.8) | 15,412 (52.5) | 5,383 (55.3) | 3,042 (56.5) |
| Female | 400 (43.2) | 5,002 (46.2) | 13,929 (47.5) | 4,357 (44.7) | 2,344 (43.5) |
| **Smoking status^a^** |  |  |  |  |  |
| Current | 149 (16.1) | 1,653 (15.3) | 4,373 (14.9) | 1,285 (13.2) | 962 (17.9) |
| Former | 319 (34.4) | 3,847 (35.5) | 10,204 (34.8) | 3,252 (33.4) | 1,775 (33.0) |
| Never | 377 (40.7) | 4,481 (41.4) | 12,173 (41.5) | 4,163 (42.7) | 2,292 (42.5) |
| Unknown | 82 (8.8) | 845 (7.8) | 2,591 (8.8) | 1,040 (10.7) | 357 (6.6) |
| **BMI^a^** |  |  |  |  |  |
| < 18.5 kg/m^2^ | 9 (1.0) | 162 (1.5) | 542 (1.8) | 188 (1.9) | 91 (1.7) |
| 18.5–24.9 kg/m^2^ | 203 (21.9) | 2,414 (22.3) | 6,757 (23.0) | 2,312 (23.7) | 1,153 (21.4) |
| 25.0–29.9 kg/m^2^ | 296 (31.9) | 3,487 (32.2) | 9,159 (31.2) | 3,123 (32.1) | 1,770 (32.9) |
| ≥ 30.0 kg/m^2^ | 279 (30.1) | 3,265 (30.2) | 8,369 (28.5) | 2,742 (28.1) | 1,767 (32.8) |
| Unknown | 140 (15.1) | 1,498 (13.8) | 4,514 (15.4) | 1,375 (14.1) | 605 (11.2) |
| **Comorbidities^b^** |  |  |  |  |  |
| Thrombocytopenia | S^c^ | 21 (0.2) | 50 (0.2) | 15 (0.2) | 14 (0.3) |
| Anemia | 153 (16.5) | 2,005 (18.5) | 6,290 (21.4) | 1,980 (20.3) | 1,205 (22.4) |
| Prior bleeding | 437 (47.1) | 4,610 (42.6) | 13,116 (44.7) | 4,477 (46.0) | 2,300 (42.7) |
| Peripheral artery disease | 32 (3.5) | 348 (3.2) | 1,048 (3.6) | 272 (2.8) | 183 (3.4) |
| Stroke/TIA | 221 (23.8) | 1,654 (15.3) | 5,828 (19.9) | 1,663 (17.1) | 849 (15.8) |
| Systemic embolism | 10 (1.1) | 326 (3.0) | 780 (2.7) | 163 (1.7) | 145 (2.7) |
| Coronary artery disease | 206 (22.2) | 2,364 (21.8) | 6,259 (21.3) | 1,929 (19.8) | 1,300 (24.1) |
| Myocardial infarction | 112 (12.1) | 1,235 (11.4) | 3,416 (11.6) | 991 (10.2) | 674 (12.5) |
| Congestive heart failure | 123 (13.3) | 1,561 (14.4) | 5,184 (17.7) | 1,530 (15.7) | 983 (18.3) |
| Hypertension | 681 (73.5) | 7,749 (71.6) | 21,113 (72.0) | 6,877 (70.6) | 4,002 (74.3) |
| CABG/PCI | 84 (9.1) | 1,008 (9.3) | 2,764 (9.4) | 822 (8.4) | 571 (10.6) |
| Pacemaker | 79 (8.5) | 746 (6.9) | 1,690 (5.8) | 792 (8.1) | 417 (7.7) |
| History of falls | 216 (23.3) | 2,667 (24.6) | 8,438 (28.8) | 2,746 (28.2) | 1,201 (22.3) |
| COPD | 221 (23.8) | 2,715 (25.1) | 7,702 (26.2) | 2,459 (25.2) | 1,356 (25.2) |
| Acute kidney injury | 43 (4.6) | 550 (5.1) | 2,134 (7.3) | 627 (6.4) | 311 (5.8) |
| Chronic kidney disease | 240 (25.9) | 2,987 (27.6) | 8,808 (30.0) | 2,659 (27.3) | 2,067 (38.4) |
| Depression | 192 (20.7) | 2,377 (22.0) | 6,952 (23.7) | 2,282 (23.4) | 1,110 (20.6) |
| Dementia or MCI | 33 (3.6) | 537 (5.0) | 1,576 (5.4) | 551 (5.7) | 185 (3.4) |
| Parkinson’s disease | S^c^ | 15 (0.1) | 53 (0.2) | 26 (0.3) | 16 (0.3) |
| Liver disease | 33 (3.6) | 486 (4.5) | 1,491 (5.1) | 571 (5.9) | 197 (3.7) |
| Diabetes mellitus | 259 (27.9) | 3,285 (30.3) | 9,387 (32.0) | 3,061 (31.4) | 1,718 (31.9) |
| **Comedications^d^** |  |  |  |  |  |
| Antidiabetic | 117 (12.6) | 1,703 (15.7) | 5,025 (17.1) | 1,537 (15.8) | 950 (17.6) |
| Antihypertensives | 752 (81.1) | 8,707 (80.4) | 23,226 (79.2) | 7,592 (77.9) | 4,642 (86.2) |
| Lipid-lowering drugs | 483 (52.1) | 5,366 (49.6) | 14,395 (49.1) | 4,807 (49.4) | 2,494 (46.3) |
| Antiarrhythmics | 28 (3.0) | 207 (1.9) | 405 (1.4) | 133 (1.4) | 128 (2.4) |
| Digoxin | 26 (2.8) | 295 (2.7) | 687 (2.3) | 145 (1.5) | 249 (4.6) |
| Antiplatelets | 419 (45.2) | 4,525 (41.8) | 12,145 (41.4) | 3,598 (36.9) | 2,523 (46.8) |
| NSAIDs | 242 (26.1) | 2,539 (23.5) | 6,192 (21.1) | 1,856 (19.1) | 1,237 (23.0) |
| Antidepressants | 161 (17.4) | 1,853 (17.1) | 5,321 (18.1) | 1,643 (16.9) | 803 (14.9) |
| Antipsychotics | 38 (4.1) | 584 (5.4) | 1,523 (5.2) | 513 (5.3) | 277 (5.1) |
| Antiepileptics | 84 (9.1) | 861 (8.0) | 2,842 (9.7) | 921 (9.5) | 533 (9.9) |
| Benzodiazepines /hypnotics | 42 (4.5) | 542 (5.0) | 1,545 (5.3) | 428 (4.4) | 265 (4.9) |
| Systemic corticosteroids | 136 (14.7) | 1,635 (15.1) | 4,471 (15.2) | 1,247 (12.8) | 873 (16.2) |
| Opioids | 307 (33.1) | 3,620 (33.4) | 10,098 (34.4) | 2,886 (29.6) | 1,968 (36.5) |
| H2 blockers | 37 (4.0) | 364 (3.4) | 1,082 (3.7) | 241 (2.5) | 229 (4.3) |
| Proton pump inhibitors | 413 (44.6) | 5,191 (47.9) | 14,853 (50.6) | 4,904 (50.3) | 2,593 (48.1) |
| Hormone replacement therapy | 11 (1.2) | 115 (1.1) | 301 (1.0) | 108 (1.1) | 45 (0.8) |
| **Cancer type** |  |  |  |  |  |
| Lung | 15 (1.6) | 177 (1.6) | 547 (1.9) | 166 (1.7) | 77 (1.4) |
| Prostate | 122 (13.2) | 1,326 (12.2) | 3,095 (10.5) | 1,235 (12.7) | 609 (11.3) |
| Colorectal | 73 (7.9) | 711 (6.6) | 1,824 (6.2) | 574 (5.9) | 331 (6.1) |
| Breast | 110 (11.9) | 1,354 (12.5) | 3,484 (11.9) | 1,132 (11.6) | 595 (11.0) |
| Hematologic | 46 (5.0) | 462 (4.3) | 1,221 (4.2) | 345 (3.5) | 231 (4.3) |
| Other cancers | 561 (60.5) | 6,796 (62.8) | 19,170 (65.3) | 6,288 (64.6) | 3,543 (65.8) |
| **Metastatic cancer** | S^c^ | 22 (0.2) | 84 (0.3) | 26 (0.3) | 11 (0.2) |
| **Cancer–related treatment** |  |  |  |  |  |
| Radiotherapy | S^c^ | 95 (0.9) | 280 (1.0) | 91 (0.9) | 57 (1.1) |
| Chemotherapy | 8 (0.9) | 126 (1.2) | 346 (1.2) | 98 (1.0) | 44 (0.8) |
| Surgery | 9 (1.0) | 72 (0.7) | 217 (0.7) | 81 (0.8) | 29 (0.5) |
| **CHA_2_DS_2_–VASc** |  |  |  |  |  |
| 2–3 | 367 (39.6) | 4,765 (44.0) | 11,570 (39.4) | 4,192 (43.0) | 2,227 (41.3) |
| 4–5 | 434 (46.8) | 4,800 (44.3) | 13,208 (45.0) | 4,289 (44.0) | 2,434 (45.2) |
| ≥ 6 | 126 (13.6) | 1,261 (11.6) | 4,563 (15.6) | 1,259 (12.9) | 725 (13.5) |
| **Modified HAS–BLED** |  |  |  |  |  |
| < 3 | 295 (31.2) | 4,002 (37.0) | 10,301 (35.1) | 3,674 (37.7) | 1,777 (33.0) |
| ≥ 3 | 632 (68.2) | 6,824 (63.0) | 19,040 (64.9) | 6,066 (62.3) | 3,609 (67.0) |
| **Dose** |  |  |  |  |  |
| Low dose | 548 (59.1) | 1,616 (14.9) | 7,070 (24.1) | 2,914 (29.9) | - |
| Standard dose | 298 (32.1) | 7,224 (66.7) | 17,702 (60.3) | 5,140 (52.8) | - |
| Unknown | 81 (8.7) | 1,986 (18.3) | 4,569 (15.6) | 1,686 (17.3) | - |

All values are expressed as n (%), unless otherwise specified.

^a^Smoking and obesity were measured in the 5 years prior to OAC initiation.

^b^Comorbidities were measured at anytime prior to OAC initiation.

^c^Cell counts < 5 are suppressed, in accordance with CPRD confidentiality policy.

^d^Comedications were measured in the year prior to OAC initiation.

Abbreviations: AF = atrial fibrillation; BMI = body mass index; CABG_PCI = coronary artery bypass surgery or percutaneous coronary intervention; CHA_2_DS_2_-VASc = congestive heart failure, hypertension, age ≥75 years, diabetes mellitus, previous stroke/TIA, vascular disease, age 65-74 years, female sex; COPD = chronic obstructive pulmonary disease; CPRD = Clinical Practice Research Datalink; HAS-BLED = uncontrolled hypertension, abnormal renal or liver function, previous stroke, bleeding history, labile international normalized ratio, age >65 years, use of NSAIDs or antiplatelets or alcohol-related disorders; MCI = mild cognitive impairment; NSAIDs = non-steroidal anti-inflammatory drugs; OAC = oral anticoagulant; SD = standard deviation; TIA = transient ischemic attack; UK = United Kingdom.

# **Table S7**. Factors associated with OAC initiation in the year following AF diagnosis in patients with cancer in the UK CPRD, 2015–2023.

|  | **Variables^a^** | **Initiators**  **(n = 56,220)** | **Non–initiators**  **(n = 16,095)** | **Crude HR**  **(95% CI)** | **Adjusted^b^ HR**  **(95% CI)** |
| --- | --- | --- | --- | --- | --- |
|  | **Clinical characteristics** |  |  |  |  |
|  | **Age categories** |  |  |  |  |
|  | 60-69 | 5,440 (9.7) | 1,451 (9.0) | 1.18 (1.09–1.28) | 1.20 (1.10–1.30) |
|  | 70-79 | 20,934 (37.2) | 4,658 (28.9) | 1.30 (1.21–1.40) | 1.33 (1.23–1.44) |
|  | ≥80 | 29,126 (51.8) | 9,743 (60.5) | 1.11 (1.03–1.20) | 1.18 (1.09–1.30) |
|  | **Male sex** | 30,188 (53.7) | 8,556 (53.2) | 1.00 (0.99–1.02) | 1.00 (0.98–1.02) |
|  | **Prior stroke/TIA** | 9,786 (17.4) | 2,806 (17.4) | 1.00 (0.98–1.03) | 1.04 (1.01–1.06) |
|  | **Prior bleeding** | 24,767 (44.1) | 7,509 (46.6) | 0.94 (0.93–0.95) | 0.95 (0.93–0.97) |
|  | **Comorbidities** |  |  |  |  |
|  | Anemia | 11,409 (20.3) | 4,662 (29.0) | 0.79 (0.78–0.80) | 0.82 (0.80–0.84) |
|  | Thrombocytopenia | 83 (0.1) | 81 (0.5) | 0.48 (0.39–0.59) | 0.52 (0.42–0.65) |
|  | Dementia | 2,753 (4.9) | 1,792 (11.1) | 0.65 (0.62–0.67) | 0.67 (0.65–0.70) |
|  | Chronic kidney disease | 16,676 (29.7) | 5,684 (35.3) | 0.89 (0.88–0.91) | 0.93 (0.91–0.95) |
|  | Hypertension | 40,308 (71.7) | 11,110 (69.0) | 1.06 (1.03–1.08) | 1.08 (1.06–1.10) |
|  | Congestive heart failure | 8,173 (14.5) | 2,441 (15.2) | 0.96 (0.94–0.98) | 0.98 (0.96–1.01) |
|  | Coronary artery disease | 11,901 (21.2) | 3,583 (22.3) | 0.97 (0.95–0.99) | 1.00 (0.97–1.02) |
|  | Diabetes | 17,570 (31.3) | 5,139 (31.9) | 0.97 (0.95–0.99) | 0.99 (0.97–1.01) |
|  | Liver disease | 2,740 (4.9) | 873 (5.4) | 0.89 (0.86–0.93) | 0.89 (0.85–0.93) |
|  | **Cancer site** |  |  |  |  |
|  | Lung | 982 (1.7) | 596 (3.7) | 0.70 (0.65–0.74) | 0.67 (0.63–0.72) |
|  | Breast | 6,675 (11.9) | 1,583 (9.8) | 1.09 (1.07–1.12) | 1.05 (1.02–1.08) |
|  | Colorectal | 3,513 (6.2) | 1,126 (7.0) | 0.94 (0.91–0.98) | 0.94 (0.90–0.97) |
|  | Prostate | 6,387 (11.4) | 1,502 (9.3) | 1.10 (1.08–1.13) | 1.06 (1.03–1.09) |
|  | Hematologic | 2,305 (4.1) | 722 (4.5) | 0.84 (0.81–0.88) | 0.84 (0.80–0.88) |
|  | **Metastatic cancer** | 146 (0.3) | 100 (0.6) | 0.68 (0.58–0.80) | 0.66 (0.56–0.77) |
|  | **Cancer treatment** |  |  |  |  |
|  | Radiotherapy | 485 (0.9) | 281 (1.7) | 0.72 (0.66–0.78) | 0.76 (0.69–0.84) |
|  | Chemotherapy | 567 (1.0) | 412 (2.6) | 0.57 (0.53–0.62) | 0.59 (0.54–0.64) |
|  | **CHA_2_DS_2_-VASc^c^** |  |  |  |  |
|  | 4–5 | 24,961 (44.4) | 6,969 (43.3) | 1.01 (0.99–1.03) |  |
|  | ≥ 6 | 7,453 (13.3) | 2,370 (14.7) | 0.95 (0.93–0.98) |  |
|  | **Modified HASBLED^d^** ≥ 3 | 38,971 (69.3) | 11,192 (69.5) | 1.00 (0.98–1.01) |  |

^a^Comorbidities were measured at any time before AF diagnosis. Cancer treatment was measured in the year before AF diagnosis.

^b^Estimates represent the association between each variable and OAC initiation, adjusted for all other variables listed in the table.

^c^Estimates for the CHA_2_DS_2_-VASc categories were calculated in a separate model.

^d^Estimate for the HASBLED score was calculated in a separate model.

Abbreviations: AF = atrial fibrillation; CI = confidence interval; CPRD = Clinical Practice Research Datalink; HR = hazard ratio; OAC = oral anticoagulant; TIA = transient ischemic attack; UK = United Kingdom.

# **Figure S1**. Flowchart of cohort formation of patients with atrial fibrillation (AF) and cancer in UK Clinical Practice Research Datalink between January 1, 2021, and December 31, 2023.

**Figure S2**. Annual incidence rates of oral anticoagulant initiation in patients with atrial fibrillation and cancer in the UK Clinical Practice Research Datalink, 2010–2023, stratified by sex. (A): active cancer; (B): overall cohort.

**Figure S3**. Annual incidence rates of oral anticoagulant initiation in patients with atrial fibrillation and cancer in the UK Clinical Practice Research Datalink, 2010–2023, stratified by HAS–BLED score. (A) active cancer; (B) overall cohort.

**Figure S4**. Annual incidence rates of oral anticoagulant initiation in patients with atrial fibrillation and cancer in the UK Clinical Practice Research Datalink, 2010–2023, stratified by bleeding history. (A) active cancer; (B) overall cohort.

**Figure S5**. Annual incidence rates of oral anticoagulant initiation in patients with atrial fibrillation and active cancer in the UK Clinical Practice Research Datalink, 2010–2023, stratified by UK nations.

**Figure S6**. Annual incidence rates of oral anticoagulant initiation in patients with atrial fibrillation and cancer (overall cohort) in the UK Clinical Practice Research Datalink, 2010–2023, stratified by UK nations.

**Figure S7**. Annual incidence rates of oral anticoagulant initiation in patients with atrial fibrillation and cancer (overall cohort) in the UK Clinical Practice Research Datalink, 2010–2023, by individual direct oral anticoagulants (DOACs) and vitamin K antagonists (VKAs).

**Figure S8**. Annual incidence rates of oral anticoagulant initiation in patients with atrial fibrillation and cancer (overall cohort) in the UK Clinical Practice Research Datalink, 2010–2023, stratified by age. (A) DOACs; (B) VKAs.

**Figure S9**. Five-year persistence with DOACs versus VKAs after first OAC initiation in patients with atrial fibrillation and cancer (overall cohort) in the UK Clinical Practice Research Datalink, 2010–2023.

**Figure S10**. Annual incidence rates of oral anticoagulant initiation in patients with atrial fibrillation and active cancer in the UK Clinical Practice Research Datalink, 2010–2023, overall and by direct oral anticoagulants (DOACs) vs vitamin K antagonists (VKAs). (A) cancer diagnosed within 1 year before AF diagnosis; (B) cancer diagnosed within 6 months before AF diagnosis, receipt of radiotherapy or chemotherapy, cancer-directed surgery, or a recorded metastatic disease codes in 6 months before AF diagnosis.
